# Optical arbitrary waveform generation (OAWG) using actively phase-stabilized spectral stitching

**DOI:** 10.1038/s41377-025-01937-4

**Published:** 2025-09-29

**Authors:** Daniel Drayss, Dengyang Fang, Alban Sherifaj, Huanfa Peng, Christoph Füllner, Thomas Henauer, Grigory Lihachev, Lennart Schmitz, Tobias Harter, Wolfgang Freude, Sebastian Randel, Tobias J. Kippenberg, Thomas Zwick, Christian Koos

**Affiliations:** 1 Institute of Photonics and Quantum Electronics (IPQ), Karlsruhe Institute of Technology (KIT), 76131 Karlsruhe, Germany; 2 Institute of Microstructure Technology (IMT), Karlsruhe Institute of Technology (KIT), 76344 Eggenstein-Leopoldshafen, Germany; 3 Teragear GmbH, 76227 Karlsruhe, Germany; 4 Institute of Radio Frequency Engineering and Electronics (IHE), Karlsruhe Institute of Technology (KIT), 76131 Karlsruhe, Germany; 5 Institute of Physics, Swiss Federal Institute of Technology Lausanne (EPFL), 1015 Lausanne, Switzerland; 6 Deeplight SA, 1025 St Sulpice, Switzerland

**Keywords:** Fibre optics and optical communications, Optoelectronic devices and components, Microwave photonics, Frequency combs

## Abstract

The conventional way of generating optical waveforms relies on in-phase and quadrature (IQ) modulation of a continuous-wave (CW) laser tone. In this case, the bandwidth of the resulting optical waveform is limited by the underlying electronic components, in particular by the digital-to-analog converters (DACs) generating the drive signals for the IQ modulator. This bandwidth bottleneck can be overcome by using a concept known as optical arbitrary waveform generation (OAWG), where multiple IQ modulators and DACs are operated in parallel to first synthesize individual spectral slices, which are subsequently combined to form a single ultra-broadband arbitrary optical waveform. However, targeted synthesis of arbitrary optical waveforms from multiple spectral slices has so far been hampered by difficulties to maintain the correct optical phase relationship between the slices. In this paper, we propose and demonstrate spectrally sliced OAWG with active phase stabilization, which permits targeted synthesis of truly arbitrary optical waveforms. We demonstrate the viability of the scheme by synthesizing optical waveforms with record-high bandwidths of up to 325 GHz from four individually generated optical tributaries. In a proof-of-concept experiment, we use the OAWG system to generate 32QAM data signals at symbol rates of up to 320 GBd, which we transmit over 87 km of single-mode fiber and receive by a two-channel non-sliced optical arbitrary waveform measurement (OAWM) system, achieving excellent signal quality. We believe that our scheme can unlock the full potential of OAWG and disrupt a wide range of applications in high-speed optical communications, photonic-electronic digital-to-analog conversion, as well as advanced test and measurement in science and industry.

## Introduction

Optical arbitrary waveforms are usually generated by sending a continuous-wave (CW) optical carrier through an in-phase and quadrature modulator (IQM), which is driven by a pair of radio frequency (RF) signals^1^. In this case, the bandwidth of the resulting optical waveform is dictated by the bandwidth of the underlying electronic components, such as the digital-to-analog converters (DACs), the driver amplifiers, and the IQMs, which are typically limited to less than 100 GHz^2–9^. This limitation can be overcome by using optical arbitrary waveform generation (OAWG) techniques^10–13^, which exploit optical frequency combs as multi-wavelength carriers for spectrally sliced signal synthesis. In this approach, the phase-locked comb tones are first modulated independently by an array of IQMs and associated DACs, and the resulting tributary signals are then merged into a single broadband optical waveform. However, while this scheme renders the bandwidth of the synthesized optical waveform independent of the bandwidth of the underlying electronic components, synthesis of truly arbitrary waveforms has so far been hindered by phase drifts among the individually generated optical tributary signals. Previous experiments thus either required a selection of measurements with coincidentally correct phase relations^10–12^ or, in the case of communication signals, non-standard data-aided processing at the receiver to compensate for the phase drifts among the tributaries at the comb-based OAWG transmitter^13^. This severely limits the viability and the application potential of spectrally sliced OAWG.

In this paper, we demonstrate an OAWG scheme that relies on active stabilization of the phases with which the various tributary signals are combined, thereby enabling the generation of truly arbitrary waveforms with good signal quality^14,15^. In a proof-of-concept experiment, we implement a four-slice phase-stabilized OAWG system, offering a record-high bandwidth of 325 GHz. Our OAWG system is carefully calibrated based on a dedicated system model, thus permitting high-fidelity waveform generation. We demonstrate the viability of the concept in a high-symbol-rate optical transmission experiment, combining phase-stabilized OAWG with non-sliced optical arbitrary waveform measurement (OAWM)^16^. Using the combined OAWG/OAWM setup, we transmit fully coherent 16QAM and 32QAM signals with symbol rates of up to 320 GBd. Moreover, we characterize the system performance at various optical signal-to-noise ratio (OSNR) levels, obtaining a maximum achievable information rate (AIR) of 1.8 Tbit/s for a single-polarization 64QAM signal. To the best of our knowledge, our work represents the first OAWG demonstration using actively phase-stabilized signal synthesis, leading to the highest bandwidth so far achieved in any OAWG experiment as well as to the highest symbol rate demonstrated for fully coherent QAM data signals, for which the pulse shape is defined digitally. We believe that our scheme can unlock the full potential of OAWG and serve a wide range of applications, such as advanced test and measurement equipment, e.g., for high-speed optical communications, high-quality signal generation in microwave and millimeter-wave photonics, or photonic-electronic digital-to-analog conversion.

## Results

### Vision and concept of OAWG transmitter

**Fig. 1:**
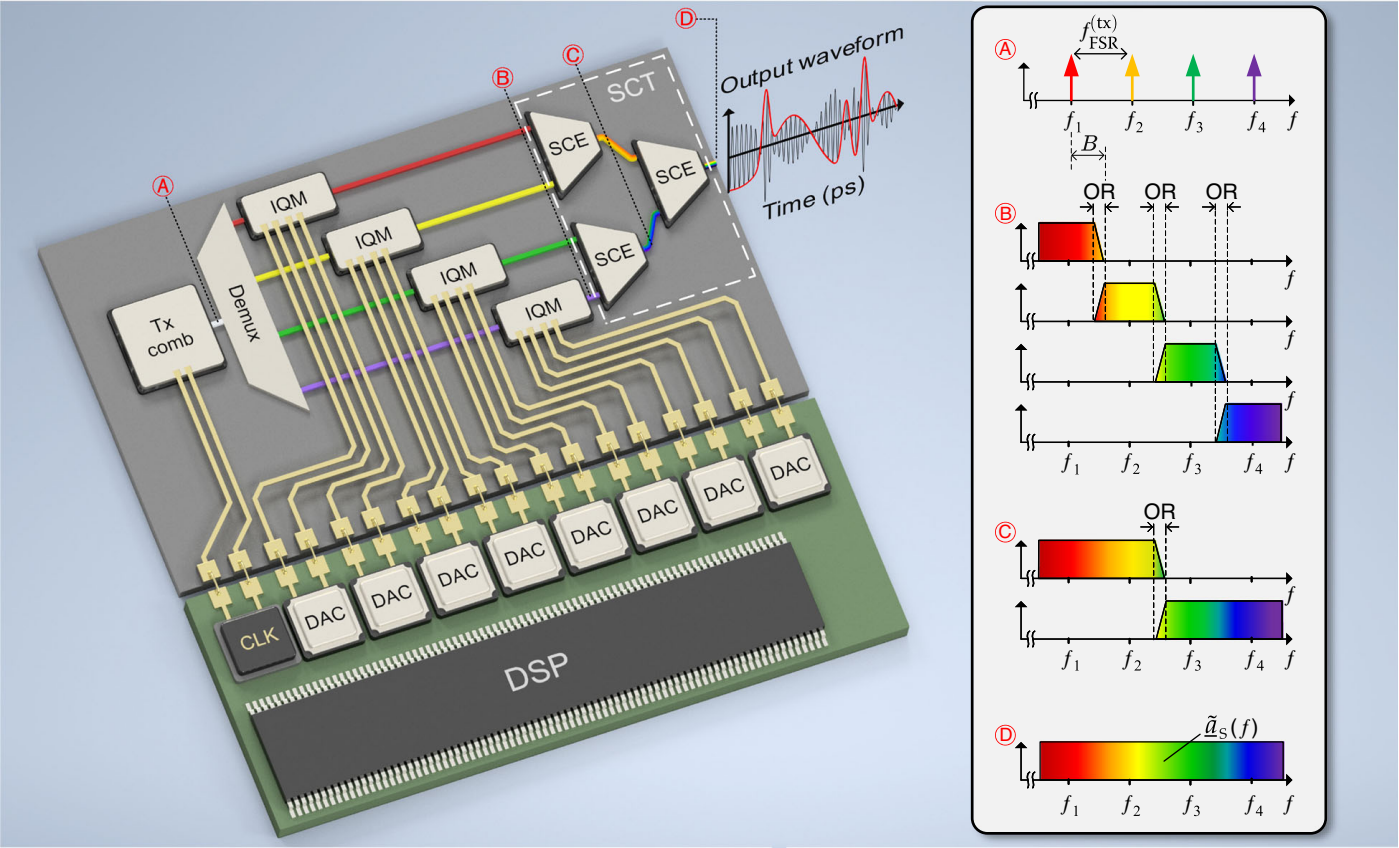
Simplified vision of a spectrally sliced OAWG system that comprises ${\boldsymbol{N}}={\mathbf{4}}$ channels with active phase stabilization and that allows to generate optical arbitrary waveforms varying on timescales of a few picoseconds. A transmitter frequency comb (Tx comb) is used to generate $N=4$ phase-locked optical tones at frequencies $f_{1}, \, f_{2}, \, f_{3},\, f_{4}$, spaced by a free spectral range (FSR) ${f}_{{\rm{FSR}}}^{{\rm{(tx)}}}$, see Inset Ⓐ. These tones are separated by a demultiplexing filter (Demux) and subsequently serve as optical carriers for in-phase and quadrature (IQ) modulation. The IQ modulators (IQMs) are electrically driven by an array of 2$N=8$ synchronized digital-to-analog converters (DACs) having each a bandwidth $B > ({f}_{{\rm{FSR}}}^{{\rm{(tx)}}}/2)$. The drive signals are calculated based on the desired output waveform using a digital signal processing (DSP) unit. The various output signals of the IQMs are combined by a binary signal-combining tree (SCT) that consists of $N - 1=3$ signal-combining elements (SCEs). Each SCE merges two spectrally adjacent tributaries, see Inset Ⓒ, where the slowly varying phase offset $\Delta\varphi(t)$ is compensated by a closed-loop control. To this end, the various tributaries are designed to exhibit a slight spectral overlap with their respective neighbor. This leads to interference within well-defined overlap regions (ORs), Insets Ⓑ and Ⓒ, which provide the feedback signals for the closed-loop control. The output signal of the last SCE corresponds to the desired optical waveform ${\underline{a}}_{{\rm{S}}}(t)$ with spectrum ${\underline{\tilde{a}}}_{{\rm{S}}}(f)$, Inset Ⓓ

**Fig. 2:**
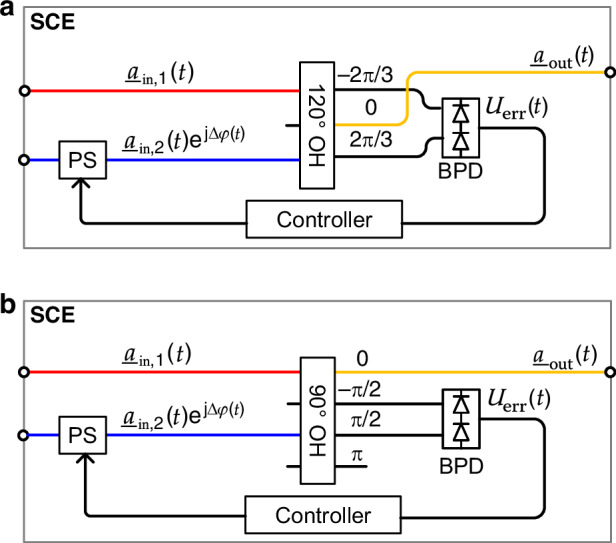
Implementation of signal-combining elements (SCEs) with closed-loop phase stabilization. **a** Conceptual setup of an SCE relying on a 120^∘^ optical hybrid (OH), which is implemented as 3 × 3 multi-mode interference (MMI) coupler with one unused input port. The MMI superimposes the input signals with relative phases of −2*π*/3, 0, and 2*π*/3 at the various output ports. Two overlapping spectral slices ${\underline{a}}_{{\rm{in}},1}(t)$ and ${\underline{a}}_{{\rm{in}},2}(t)\exp \left({\rm{j}}\Delta \varphi (t)\right)$ with phase error $\Delta\varphi(t)$ are combined at the “zero-phase” port, leading to the optical output signal ${\underline{a}}_{{\rm{out}}}(t)$. The error signal $U_{\rm{err}}(t)$ is generated by connecting the remaining two output ports of the 120^∘^ OH, which are associated with relative phases of −2*π*/3 and 2*π*/3 between the input signals, to a low-speed balanced photodetector (BPD). Upon low-pass filtering, the resulting BPD output signal $U_{\rm{err}}(t)$ is essentially proportional to phase error $\Delta\varphi(t)$ for a linear approximation close to the desired operating point $\Delta\varphi=0$ see Eq. (6) and Section S1.1 in Supplementary information 1. A proportional-integral (PI) controller is used to drive the phase shifter (PS) and to compensate for the measured phase error $\Delta\varphi(t)$. **b** Alternative SCE implementation using a 90^∘^ OH as passive combiner, e.g., implemented as a 4 × 4 MMI with two unused input ports. The targeted output signal is found again at the “zero-phase” port, and the error signals are derived by a BPD connected to the “ − *π*/2” and the “*π*/2” ports. The remaining output port “*π*” is unused in this implementation

**Fig. 3:**
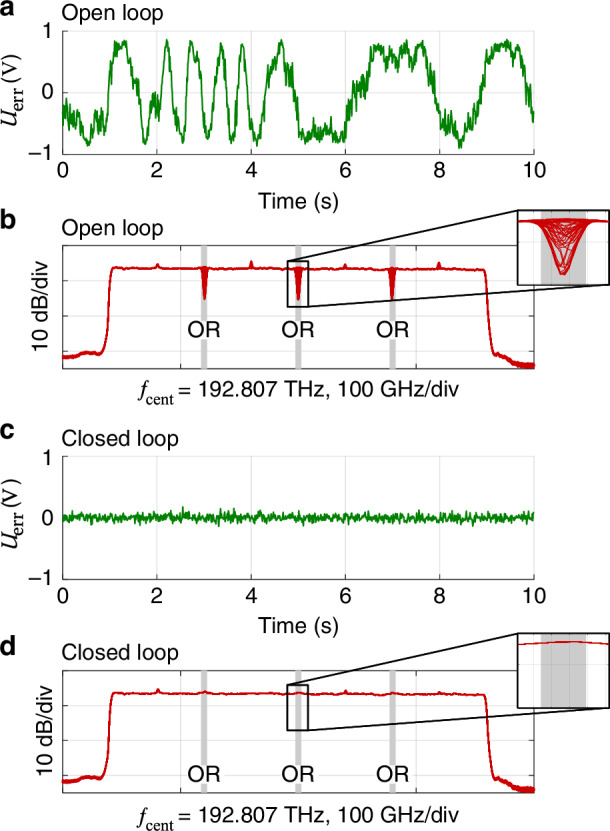
Experimental verification of the actively phase-stabilizing signal-combining tree (SCT) based on 90^∘^ optical hybrids as passive combiners, see Fig. 2(b). **a** Exemplary error signal $U_{\rm{err}}(t)$ measured for a signal-combining element (SCE) with deactivated control loop. Random variations of the error signal are observed as the phase of the different spectral slices drifts randomly. **b** Overlay of 50 optical spectra recorded with deactivated control loop. The random drift of the relative phase $\Delta \varphi(t)$ between adjacent spectral slices leads to randomly occurring spectral dips due to constructive and destructive interference in the overlap regions (ORs, marked in gray). Note that the depth of the measured dips is limited due to the non-zero resolution bandwidth (RBW = 2.48 GHz) of the optical spectrum analyzer (AQ6317B, Ando Electric Co., Ltd, now Yokogawa Electric Crop, Tokyo, Japan). **c** Residual error signal $U_{\rm{err}}(t)$ measured when the phase control is activated. **d** Overlay of 50 optical spectra recorded with activated control loop. The relative phases of all spectral slices are aligned and stabilized, and no spectral dips can be found in the ORs (marked in gray)

A simplified vision of an exemplary spectrally sliced OAWG system with active phase stabilization is illustrated in Fig. 1. The example relies on $N=4$ tributary signals, which are generated by in-phase and quadrature modulation of four phase-locked tones of an optical frequency comb (Tx comb) and which are merged in a binary tree of $N-1=3$ signal-combining elements (SCEs), each comprising feedback-based phase stabilization. The $N=4$ comb tones at frequencies $f_1,\, f_2,\, f_3,\, f_4$, spaced by a free spectral range (FSR) ${f}_{{\rm{FSR}}}^{{\rm{(tx)}}}$, are illustrated in Inset Ⓐ of Fig. 1. Importantly, the FSR of the Tx comb source is synchronized to the clock (CLK) of the DAC array to achieve full coherence among all subsequently generated spectral slices. This can, e.g., be accomplished by generating the Tx comb via electro-optic modulation of a CW laser tone where the modulator’s driver signals are synchronized to the electronic clock^17,18^ or by RF synchronized pulsed solid-state lasers^19,20^. The generated frequency comb is then fed to a demultiplexing filter (DEMUX), which may be implemented, e.g., as a wavelength-selective switch (WSS), an arrayed waveguide grating^21,22^, or a bank of ring filters^23,24^, and which separates the various comb tones for subsequent IQ modulation. The IQMs are electrically driven by an array of 2 $N=8$ synchronized DACs, which are connected to a digital signal processing (DSP) unit that calculates the various IQ drive signals from the targeted output waveform. Merging of the $N$ tributaries in the signal-combining tree (SCT) finally produces the targeted broadband arbitrary optical waveform ${\underline{a}}_{{\rm{S}}}(t)$ with spectrum, ${\underline{\tilde{a}}}_{{\rm{S}}}(f)$ see Inset Ⓓ of Fig. 1. Within the SCT, each of the $N-1=3$ SCEs combines two adjacent tributaries, see Inset Ⓒ in Fig. 1, where the slowly varying phase offset $\Delta\varphi(t)$ is minimized by a closed-loop control. To this end, the various tributaries are deliberately designed to exhibit a slight spectral overlap with their respective neighbor. This leads to interference within well-defined overlap regions (ORs), see Insets Ⓑ and Ⓒ in Fig. 1, which provides the feedback signals for the closed-loop control, refer to Figs. 2 and 3 and the discussion thereof in “Actively phase-stabilizing signal-combining element” below for details. The bandwidth of the generated optical arbitrary waveform exceeds that of the individual DACs by a factor of ~2$N$, allowing the synthesis of waveforms with bandwidths of hundreds of GHz that vary on single-digit picosecond time scales.

#### System model

For high-fidelity OAWG using the scheme illustrated in Fig. 1, the characteristics of the various system components must be known and considered in the design of the drive signals that are fed to the various IQMs. In this section, we derive a linear system model for a generalized OAWG transmitter featuring an array of $N$ IQMs and $N$ corresponding spectrally sliced tributaries. The concept of the active phase stabilization of the SCE is explained in the subsequent section “Actively phase-stabilizing signal-combining element.” In a first step, we express the spectrum ${\underline{\tilde{a}}}_{{\rm{S}}}(f)$ of the arbitrary optical output waveform ${\underline{a}}_{{\rm{S}}}(t)$ as a superposition of *N* individual tributaries ${\underline{a}}_{{\rm{S}},\nu }(t)$ with spectra ${\underline{\tilde{a}}}_{{\rm{S}},\nu }(f)$, $v=1,\, \ldots,\, N$,1where the transformation symbol () is used to denote a transfer between the time and the frequency domain via a Fourier transform. Note that the tributary signals ${\underline{a}}_{{\rm{S}},\nu }(t)$ refer to the output of the SCT, point Ⓓ in Fig. 1, such that the transfer characteristics of all SCEs and other system elements are already included. Each of the time-domain tributary signals ${\underline{a}}_{{\rm{S}},\nu }(t)$ can be expressed by a complex-valued envelope ${\underline{A}}_{{\rm{S}},\nu }(t)$ and a corresponding comb-tone, acting as a carrier at frequency *f*_*ν*_,2

The complex-valued envelope ${\underline{A}}_{{\rm{S}},\nu }(t)$ of each of the optical tributary signals is related to the corresponding electrical drive signals *I*_*ν*_(*t*) and *Q*_*ν*_(*t*) of the various IQMs by equivalent baseband transfer functions ${\underline{\tilde{H}}}_{\nu }^{{\rm{(I)}}}\,(f)$ and ${\underline{\tilde{H}}}_{\nu }^{{\rm{(Q)}}}\,(f)$, that combine all optical and electrical transfer functions of the respective signal path,

3 $${\underline{\tilde{A}}}_{{\rm{S}},\nu}(f)={\underline{\tilde{H}}}_{\nu}^{{\rm{(I)}}}(f){\tilde{I}}_{\nu }(f)+{\rm{j}}\,{\underline{\tilde{H}}}_{\nu }^{{\rm{(Q)}}}(f){\tilde{Q}}_{\nu }(f)$$

In this relation, ${\tilde{I}}_{\nu }(f)$ and ${\tilde{Q}}_{\nu }(f)$ are the Fourier spectra of the electrical IQ drive signals $I_{v}(t)$ and $Q_{v}(t)$, respectively. Note that for the derivation of the above relation, we assumed that the various IQMs are biased at the zero-transmission point, that the in-phase and quadrature components have an ideal 90^∘^ phase relationship (factor j), and that all drive signals are sufficiently small such that a linear approximation of the electro-optic (EO) transfer function of the Mach-Zehnder modulator (MZM) can be used.

The IQ drive signals $I_{v}(t)$ and $Q_{v}(t)$ finally need to be designed to produce the targeted arbitrary optical waveform ${\underline{a}}_{{\rm{S}}}^{{\rm{(tar)}}}(t)$ at the SCT output, which is accomplished in two steps: First, we calculate the targeted envelope spectra ${\underline{\tilde{A}}}_{{\rm{S}},\nu }^{{\rm{(tar)}}}(f)$ of the various tributary signals, $v=1,\, \ldots,\, N$ from the targeted optical waveform ${\underline{a}}_{{\rm{S}}}^{{\rm{(tar)}}}(t)$. In a second step, we use appropriate signal pre-distortion to conceive IQ drive signals that compensate for the transfer characteristics of the various electrical and optical components along the signal path to the SCT output. For the first step, we multiply the targeted optical spectrum ${\underline{\tilde{a}}}_{{\rm{S}}}^{{\rm{(tar)}}}(f)$ by a series of slice-specific real-valued optical window functions $w_\nu(f)$, $\nu=1,\, \ldots,\, N$, to obtain the targeted spectral envelopes ${\underline{\tilde{A}}}_{{\rm{S}},\nu }^{{\rm{(tar)}}}(f)$ that need to be modulated onto the corresponding comb tones,

4 $${\underline{\tilde{A}}}_{{\rm{S}},\nu }^{{\rm{(tar)}}}(f-{f}_{\nu })={\underline{\tilde{a}}}_{{\rm{S}},\nu }^{{\rm{(tar)}}}(f)={w}_{\nu }(f)\times {\underline{\tilde{a}}}_{{\rm{S}}}^{{\rm{(tar)}}}(f)$$

Note that the optical bandwidth of each window function $w_{\nu}(f)$ in Eq. (4) is chosen slightly bigger than the FSR ${f}_{{\rm{FSR}}}^{{\rm{(tx)}}}$ such that the spectra ${\underline{\tilde{a}}}_{{\rm{S}},\nu }^{{\rm{(tar)}}}(f)$ of adjacent tributary signals exhibit the desired spectral overlap, see OR in Inset Ⓑ of Fig. 1, and add up to the desired amplitude within the corresponding overlap region, i.e., $\mathop{\sum }\nolimits_{\nu = 1}^{N}{w}_{\nu }(f)=1$ for all frequencies within the bandwidth of the targeted signal. In the experimental demonstration discussed below, we use an overlap region with a bandwidth of 3–5 GHz, within which the window function $w_{\nu}(f)$ either decays linearly, see Inset Ⓑ of Fig. 1, or has a constant value of 0.5, see Fig. S1 of Supplementary information 1. Based on the targeted spectral envelopes ${\underline{\tilde{A}}}_{{\rm{S}},\nu }^{{\rm{(tar)}}}(f)$, we then derive the drive signals of the various IQMs by appropriate pre-distortion based on the respective transfer functions ${\underline{\tilde{H}}}_{\nu }^{{\rm{(I)}}}(f)$ and ${\underline{\tilde{H}}}_{\nu }^{{\rm{(Q)}}}(f)$, thus, compensating for the frequency response of the DAC-array, the IQMs, the electrical and the optical amplifiers, and the optical propagation path through the SCT. To this end, we first measure all baseband transfer functions ${\underline{\tilde{H}}}_{\nu }^{{\rm{(I)}}}(f)$, ${\underline{\tilde{H}}}_{\nu }^{{\rm{(Q)}}}(f)$, in a dedicated calibration measurement, see Supplementary information 1 Section S2 for a detailed description of the calibration procedure. We then make use of the symmetry relation for spectra of real-valued signals ${\tilde{I}}_{\nu }(f)={\tilde{I}}_{\nu }^{* }(-f)$ and ${\tilde{Q}}_{\nu }(f)={\tilde{Q}}_{\nu }^{* }(-f)$, and expand Eq. (3),

5 $$\left[\begin{array}{c}{\underline{\tilde{A}}}_{{\rm{S}},\nu }^{{\rm{(tar)}}}(f)\\ {\underline{\tilde{A}}}_{{\rm{S}},\nu }^{{\rm{(tar)}}* }(-f)\end{array}\right]=\left[\begin{array}{cc}{\underline{\tilde{H}}}_{\nu }^{{\rm{(I)}}}(f)&{\rm{j}}\,{\underline{\tilde{H}}}_{\nu }^{{\rm{(Q)}}}(f)\\ {\underline{\tilde{H}}}_{\nu }^{{\rm{(I)}}* }(-f)&-{\rm{j}}\,{\underline{\tilde{H}}}_{\nu }^{{\rm{(Q)}}* }(-f)\end{array}\right]\left[\begin{array}{c}{\tilde{I}}_{\nu }(f)\\ {\tilde{Q}}_{\nu }(f)\end{array}\right]$$

The spectra ${\tilde{I}}_{\nu }(f)$ and ${\tilde{Q}}_{\nu }(f)$ of the pre-distorted IQ drive signals *I*_*ν*_(*t*) and *Q*_*ν*_(*t*) are then obtained by inverting Eq. (5).

#### Actively phase-stabilizing signal-combining element

A key aspect for the OAWG concept depicted in Fig. 1 is the actively phase-stabilized superposition of two slightly overlapping spectrally adjacent slices by the SCE. This is not only essential for setups relying on fiber-pigtailed components, which are subject to significant phase variations due to vibrations and thermal drifts, but also for integrated systems, where a closed-loop phase control allows for stable high-fidelity signal generation independent of thermal drifts within the underlying photonic integrated circuit (PIC)^25^ — just like a bias control on integrated MZMs or IQMs^26–28^. Each SCE contains a passive combiner with multiple output ports, e.g., a 120^∘^ optical hybrid as shown in Fig. 2a or a 90^∘^ optical hybrid as shown in Fig. 2b. For the experiments discussed below, we relied on a 90^∘^ optical hybrid, which was readily available as a fiber-pigtailed component. The desired optical signal ${\underline{a}}_{{\rm{out}}}(t)$ is then obtained at one of the output ports of the passive combiner, marked yellow in Fig. 2a, b, while two of the other ports are connected to a low-speed balanced photodetector (BPD) to generate an error signal $U_{\rm{err}}(t)$ that is used for feedback-based compensation of the underlying phase error $\Delta \varphi(t)$, see Supplementary information 1 Section S1 for a more detailed mathematical description. The error signal results from interference of the overlapping spectral components of neighboring tributaries, see marked overlap regions (ORs) in Insets Ⓑ and Ⓒ of Fig. 1. Under the assumption that the average optical powers of the combined tributaries within the overlap regions are constant and that the system is close to the desired operating point $\Delta \varphi=0$, a linear approximation, which renders the error signal $U_{\rm{err}}(t)$ proportional to the phase error $\Delta\phi(t)$, can be used,

6 $${U}_{{\rm{err}}}(t)\propto \sin \left(\Delta \varphi (t)\right)\approx \Delta \varphi (t)$$

The error signal $U_{\rm{err}}(t)$ is then fed to controller, which drives a phase shifter (PS) at one of the optical input ports, Fig. 2a and b. Note that, depending on the insertion loss of each SCE and the number of spectral slices combined, an optical amplifier at the output of the SCT might be required to compensate for the unavoidable optical loss that is introduced by the rather simple wavelength-agnostic SCE implementations shown in Fig. 2a and b. In this respect, the 120^∘^ optical hybrid is preferred as it does not waste power to an unused port, denoted by “*π*” in Fig. 2b, and since it can be easily implemented as a symmetrical 3 × 3 multi-mode interference (MMI) coupler. More advanced designs of SCEs, exploiting, e.g., wavelength-dependent PIC elements such as arrayed waveguide gratings could mitigate optical losses further.

To verify the viability of the SCE, we use fiber-optic components and an implementation based on a 90^∘^ optical hybrid, according to Fig. 2b. We record an exemplary error signal $U_{\rm{err}}(t)$ in the open-loop as well as in the closed-loop configuration, see Fig. 3a and c, respectively. As expected, using fiber optic components leads to a significant drift of the optical phase within 10 s if the active phase stabilization is turned off. As a result, we observe randomly occurring destructive or constructive interference within the three overlap regions of the overall output signal, leading to a random variation of the BPD’s output voltage between approximately −1 V and +1 V, Fig. 3a. The randomly occurring destructive or constructive interference is visualized in Fig. 3b, where 50 superimposed optical spectra of the overall output waveform ${\underline{a}}_{{\rm{out}}}(t)$ are shown and where strong random dips are observed in the three overlap regions. By closing the control loop, the error signal $U_{\rm{err}}(t)$, Fig. 3c, and consequently the phase error $\Delta\varphi(t)$ are minimized. As a result, all superimposed tributaries interfere constructively, and the spectral dips disappear as can be seen from the 50 superimposed spectra shown in Fig. 3d. Note that our current experiments rely on a piezo-based fiber stretcher (FPS-003, General Photonics, now part of Luna Innovations) with a tuning range of 55*π*. When used with extended fiber-based setups, the fiber-stretcher typically reaches its limit within a few tens to a few 100s of seconds, which triggers an automatic reset of the underlying digital proportional-integral (PI) controller. This undesirable reset can be avoided in future implementations by using an endless phase shifter instead of the fiber-stretcher^29^ or by integrating the system, such that a simple phase shifter with finite tuning range is sufficient^30–32^.

### 320 GBd transmission experiment

#### Experimental setup

**Fig. 4:**
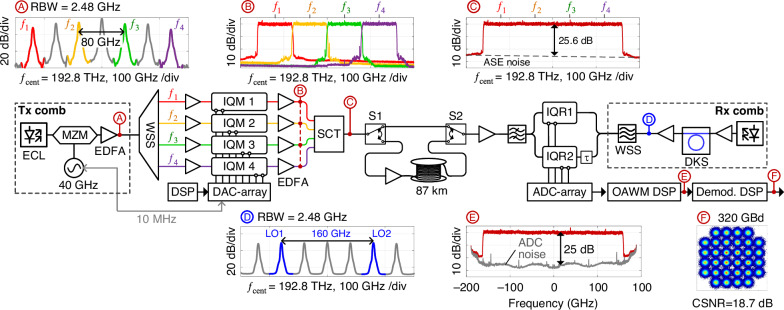
Experimental setup of our OAWG transmission experiment and exemplary measurement results taken at various points Ⓐ...Ⓕ. The transmitter comb (Tx comb, Point Ⓐ) is generated by modulating a CW tone emitted by an external-cavity laser (ECL). The resulting Tx comb is amplified by an erbium-doped fiber amplifier (EDFA), and individual tones $f_{1}, \,f_{2}, \,f_{3},\, f_{4}$ are selected by a wavelength-selective switch (WSS) to serve as carriers for IQ modulation. The drive signals for the IQ modulators (IQM1,..., IQM4) are calculated by offline digital signal processing (DSP) and generated by a DAC array (Keysight M8194A) that is RF-synchronized to the Tx comb generator. A phase-stabilizing signal-combining tree (SCT) combines all tributaries, Point Ⓑ, thus forming the output waveform ${\underline{a}}_{{\rm{S}}}(t)$, Point Ⓒ. The generated waveform is measured by a two-channel non-sliced OAWM receiver—either directly (upper position of switches S1 and S2) or after transmission through an 87 km-long fiber link (lower switch positions). The OAWM system uses two IQ receivers (IQRs) that are fed by the received waveform and by time-delayed copies of the Rx comb, comprising two tones (LO1, LO2, Inset Ⓓ) derived from a dissipative Kerr soliton (DKS) comb. The photocurrents of the IQRs are digitized by an ADC array (Keysight UXR series oscilloscope) and are used to reconstruct the received waveform via the OAWM DSP, Point Ⓔ. The reconstructed waveform is then demodulated (Demod. DSP) to retrieve the transmitted data, Point Ⓕ. Inset Ⓐ: Optical spectrum of Tx comb. Note that the displayed spectral width of the individual comb-tones is dictated by the rather large resolution bandwidth (RBW) of the spectrum analyzer that was used for the measurement (RBW = 2.48 GHz). Inset Ⓑ: High-resolution (RBW = 100 MHz) optical spectra of individual the tributary signals. Inset Ⓒ: High-resolution (RBW = 100 MHz) optical spectrum of 320 GBd 16QAM signal. Inset Ⓓ: Optical spectrum of the Rx comb (RBW = 2.48 GHz) that is used for OAWM. Inset Ⓔ: Spectrum of reconstructed 320 GBd 16QAM waveform obtained from the OAWM receiver in the optical back-to-back configuration (RBW = 100 MHz). Inset Ⓕ: Constellation diagram and constellation signal-to-noise ratio (CSNR) for an exemplary 320 GBd 32QAM signal measured in back-to-back configuration

To demonstrate the viability of the scheme described in the section above, we perform a proof-of-concept experiment in which we transmit and receive broadband optical communication signals with symbol rates of up to 320 GBd by combining phase-stabilized OAWG with non-sliced OAWM, see Fig. 4 for the associated experimental setup. The OAWG subsystem relies on a transmitter frequency comb (Tx comb), which is generated by modulating a CW laser tone using an MZM driven by a 40 GHz sinusoidal. The Tx comb is subsequently amplified by an erbium-doped fiber amplifier (EDFA), and four phase-locked tones at frequencies $f_{1}, \;f_{2}, \;f_{3}, \,{\rm{and}}\,f_{4}$ are selected by a WSS to serve as carriers for spectrally sliced signal synthesis. We measure the spectrum of the Tx comb at point Ⓐ of Fig. 4, see the corresponding inset. The optical carrier-to-noise ratio (OCNR) of all comb lines exceeds 30 dB, measured with respect to the standard reference bandwidth of 12.5 GHz, which corresponds to a wavelength interval of 0.1 nm at a center wavelength of λ = 1550 nm. The isolated optical carriers are amplified and fed to an array of four IQ-modulators (IQM 1 … IQM 4), which are electrically driven by an overall eight DAC outputs (DAC array) of two arbitrary waveform generators (Keysight M8194A), synchronized to the Tx-comb via a 10 MHz reference clock. The output signals of the IQMs are amplified (point Ⓑ in Fig. 4) and combined by an SCT that ensures stable phase relationships among all superimposed tributaries.

The electrical signals *I*_*ν*_(*t*) and *Q*_*ν*_(*t*) driving the various IQMs are pre-distorted based on Eq. (5), using the measured transfer functions ${\underline{\tilde{H}}}_{\nu }^{{\rm{(I)}}}(f)$ and ${\underline{\tilde{H}}}_{\nu }^{{\rm{(Q)}}}(f)$ that comprise the characteristics of the DAC array, the IQMs, and the electrical and optical amplifiers. The drive signals are clipped to a peak-to-average power ratio (PAPR) of 10 dB to reduce the impact of quantization noise of the DACs. Insets Ⓑ and Ⓒ of Fig. 4 depict the optical power spectra of the four tributaries and of the associated merged output waveform, for a 320 GBd 16QAM signal. We use root-raised cosine (RRC) pulse shapes with a roll-off of $\rho=0.01$, leading to a spectrally flat power spectrum, which is nicely reproduced by the directly measured optical spectrum in Insets Ⓑ and Ⓒ of Fig. 4, thus confirming the viability of our signal-generation and pre-distortion scheme. From the spectrum shown in Inset Ⓒ of Fig. 4, we estimate the in-band amplified spontaneous emission (ASE) noise level caused by the various EDFAs by interpolating the out-of-band noise, indicated by a dashed line in Inset Ⓒ of Fig. 4. Note that the overall in-band noise exceeds the pure ASE noise as it additionally comprises DAC noise, noise from electrical amplifiers, as well as distortions such as remaining IQ imbalance. We measure the overall transmitter noise and distortions by generating single-sideband signals and estimate the signal-to-noise-and-distortion ratio (SNDR) to be in the range of 22 dB for a 320 GBd optical waveform, see Supplementay information 1 Section S2.2 for details.

The generated optical signal is then either fed directly to the OAWM receiver in an optical back-to-back configuration, corresponding to the upper position of switches S1 and S2 in Fig. 4, or it is first sent through a booster amplifier and transmitted over an 87 km-long fiber link associated with the lower switch positions. At the receiver, we rely on a two-channel non-sliced OAWM system, see in ref.^16^ for details on the underlying receiver concept. The OAWM receiver uses a free-running dissipative Kerr soliton (DKS) comb (Rx comb) as a multi-wavelength local oscillator (LO)^33^, from which we extract two phase-locked tones spaced by ~160 GHz by means of a second WSS. The spectrum of the Rx comb is depicted in Inset Ⓓ of Fig. 4. Both comb lines have an OCNR of 30 dB with respect to the standard reference bandwidth of 12.5 GHz. For high-fidelity signal reconstruction, we calibrate the OAWM receiver using a known optical reference waveform generated by a femtosecond mode-locked laser, see in refs. ^16,34^ for details. The power spectrum of the reconstructed waveform measured at point Ⓔ of Fig. 4 is shown in the corresponding inset. We additionally include the power spectral density of analog-to-digital converter (ADC) noise, measured after applying the OAWM DSP (gray trace, Inset Ⓔ of Fig. 4), which is approximately 25 dB below the signal—comparable to the impairments by Tx ASE noise. From the waveform reconstructed by the OAWM receiver, we finally recover the constellation diagram of the 320 GBd 32QAM data signal shown in Inset Ⓕ of Fig. 4, which leads to a signal-to-noise ratio (CSNR) of CSNR_dB_ =  $18.7\,{\rm{dB}}$. The CSNR considers not only noise but also linear and nonlinear distortions and corresponds to the square of the reciprocal of the error vector magnitude (EVM) normalized to the average signal power^35^, ${{\rm{CSNR}}}_{{\rm{dB}}}=10{\log }_{10}\left(1/{{\rm{EVM}}}_{{\rm{a}}}^{2}\right)$, see Section S3 of Supplementary information 1 for details.

#### Transmission demonstrations at different symbol rates

**Fig. 5:**
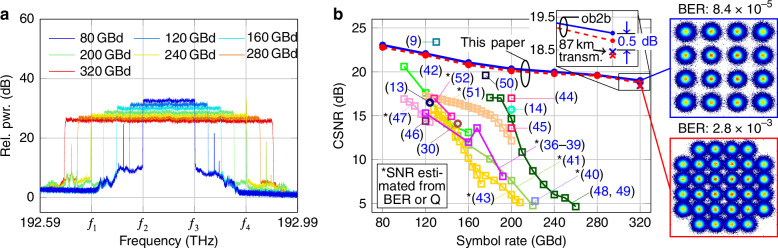
Generation and measurement of quadrature amplitude modulation (QAM) signals with symbol rates of up to 320 GBd using OAWG and OAWM techniques, and comparison to competing approaches. **a** Optical spectra of 16QAM signals with symbol rates ranging from 80 GBd to 320 GBd generated using spectrally sliced OAWG and measured using a high-resolution optical spectrum analyzer (AP2060, Apex Technologies, Marcoussis, France, resolution bandwidth 100 MHz). For better comparison, all spectra are normalized to the out-of-band amplified spontaneous emission (ASE) noise level. We indicate the frequencies $f_{1}, \;f_{2}, \;f_{3}, f_{4}$ of the Tx comb, for which a constant free spectral range (FSR) is maintained throughout the experiment. **b** Constellation signal-to-noise ratio (CSNR) as a function of the symbol rate for 16QAM (blue dots and solid blue line) and 32QAM signals (red dots and red dashed line)—measured in an optical back-to-back (ob2b) configuration and after transmission over 87 km of single-mode fiber (blue and red cross, see inset). The results are compared to other high-symbol-rate optical signaling experiments that rely on single digital-to-analog converters (DACs)^9,46,47^, or photonic-electronic^13,14,30^ (circular markers) or purely electronic^36–45,48–52^ (square markers) multiplexing techniques. References ^40,43,51^ demonstrate pulse-amplitude modulation (PAM) signaling, whereas the other publications show QAM signals. References ^41–43^ and^48–51^ use the commercially available signal generators Keysight M8199A and M8199B, respectively, which rely two time-interleaved DAC channels. Insets: Exemplary constellation diagrams for 16QAM and 32QAM 320 GBd signals and measured bit-error ratio (BER) obtained for the optical back-to-back configuration

In a first experiment, we use the system described in Fig. 4 to generate and receive 16QAM and 32QAM waveforms with symbol rates ranging from 80 GBd to 320 GBd—either in an optical back-to-back configuration or after propagating through the 87 km fiber link. Figure 5a shows the synthesized spectra of data signals of various symbol rates measured using a high-resolution optical spectrum analyzer (AP2060, Apex Technologies, Marcoussis, France, RBW = 100 MHz) directly at the OAWG transmitter output, i.e., at Point Ⓒ in Fig. 4. The frequencies *f*_1_, *f*_2_, *f*_3_, *f*_4_ of the four optical carriers are also indicated in Fig. 5a. Note that, due to limitations of the underlying RF components, the FSR of the Tx comb was kept constant throughout the experiment such that lower symbol rates resulted in only partially filled or even empty spectral slices. Figure 5b shows the CSNR obtained for various symbol rates measured in the optical back-to-back configuration for 16QAM signals (blue dots, solid line) and for 32QAM signals (red dots, dashed line). For the highest symbol rate of 320 GBd, both the 16QAM and the 32QAM signals were sent over the 87 km fiber link. After applying digital dispersion compensation, we find a CSNR penalty of 0.5 dB compared to the optical back-to-back configuration, see red and blue cross on the right of Fig. 5b and in the corresponding inset. Compared to the most advanced competing high symbol-rate QAM and PAM signaling experiments^9,13,14,30,36–52^ relying on optical^13,14,30^, or electrical^36–45,48–52^ multiplexing techniques, our experiments show significant advantages regarding the symbol rates and the CSNR, see additional data points and corresponding references in Fig. 5b. Specifically, as the spectral multiplexing is done in the optical domain, our approach does not suffer from performance degradation for symbol rates above 200 GBd, as typically observed for the purely electronic approaches^9,36–52^. The OAWG approach hence renders the achievable optical bandwidth independent of the bandwidth of the underlying electronic components. Note also that the use of demultiplexing filters for separating the frequency comb tones in combination with the active phase stabilization and the accurate transmitter calibration avoids the formation of spectral images that have been observed in other optical multiplexing techniques that, e.g., rely on optical time or phase interleaving^30,31^.

#### Transmission performance at different optical signal-to-noise ratio (OSNR) levels

**Fig. 6:**
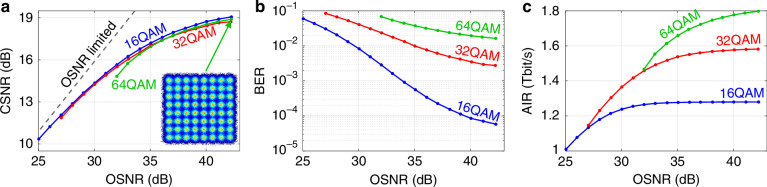
Measurement of 16QAM, 32QAM, and 64QAM signals at a symbol rate of 320 GBd at various optical signal-to-noise ratio (OSNR) levels. The OSNR is measured in a single polarization with respect to the standard reference bandwidth of 12.5 GHz, thus only including ASE noise that is co-polarized with the signal under test. **a** Constellation signal-to-noise ratio (CSNR) as a function of the OSNR for various modulation formats. The inset shows an exemplary 64QAM constellation diagram. **b** Bit-error ratio (BER) before applying forward-error correction (FEC) vs. OSNR. **c** Achievable information rate (AIR) vs. OSNR. The AIR was calculated according to Eq. (7) based on the normalized generalized mutual information (NGMI), which was estimated form the constellation diagrams

In a second experiment, we operate the system in the optical back-to-back configuration to synthesize 320 GBd QAM signals, which are then loaded with additional ASE noise to investigate the system performance at various OSNR levels, see Fig. S5 in Supplementary information 1 for the corresponding experimental setup. Note that the generated optical waveform only occupies a single polarization, and we hence only consider co-polarized ASE noise to specify OSNR levels, using the standard reference bandwidth of 12.5 GHz (0.1 nm at *λ* = 1550 nm). Figure 6a shows the measured CSNR as a function of the OSNR for various modulation formats. The dashed gray line indicates the OSNR-limited signal-to-noise ratio, obtained by considering the noise power within the entire signal bandwidth. The CSNR saturates at ~19 dB for high OSNR values, consistent with the back-to-back measurements shown in Fig. 5b, which were acquired at an OSNR level of ~40 dB. An exemplary constellation diagram of a 320 GBd 64QAM signal is shown as an inset of Fig. 6a.

Based on the OSNR measurements, we further evaluate the bit-error ratio (BER) before forward error correction, Fig. 6b, as well as the achievable information rate (AIR) Fig. 6c. The AIR is calculated based on the normalized generalized mutual information (NGMI)^53^, which is estimated from the constellation diagrams. For single-polarization QAM signals with uniform symbol probabilities and blind DSP, Eq. (1) in ref.^49^ simplifies to

7 $${\rm{AIR}}=m\,\,R\,\,{\rm{NGMI}}$$

where *R* = 320 GBd is the symbol rate and *m* is the number of bits per symbol, e.g., *m* = 4 for 16QAM, or *m* = 6 for 64QAM. As can be seen from Fig. 6c, an AIR of up to 1.8 Tbit/s is achieved for the single-polarization 320 GBd 64QAM signal at the maximum OSNR of 42 dB, which is obtained without noise loading. Note that uniform QAM signals cannot leverage the full transmission capacity and that the AIR can be further increased by probabilistic constellation shaping^54^.

## Discussion

### System performance and limitations

Our OAWG transmitter and the associated OAWM receiver offer attractive performance advantages not only in terms of the achievable bandwidth but also in terms of the overall signal quality, while still leaving room for further improvement. The dominant noise sources in our current system are ASE noise of the various EDFAs, DAC noise, ADC noise, and the limited OCNR of the Tx and Rx comb. The SNDR can hence be increased, e.g., by including an optical bandpass filter after each IQM to suppress ASE noise, or by pre-emphasizing the high-frequency components via a programmable optical filter that compensates the RF frequency responses of the DAC-array and the IQMs in the optical domain^9,48–50^. The receiver noise may be further reduced by increasing the channel count of the OAWM system and by relying on a larger number of lower-speed ADCs, see ref. ^34^ for a more detailed discussion. Similarly, the OAWG performance can be improved by using more DAC channels or by adapting the FSR of the Tx comb to the symbol rate. Specifically, for a fixed FSR as used in our current implementation, reduced symbol rates lead to spectral slices that are only partially filled or even empty, see Fig. 5a, while all active DAC channels and associated IQMs still run at the maximum RF bandwidth of 40 GHz. This leads to noticeable impairments through bandwidth limitations and limited SNDR of the various drive signals, which can be mitigated by using spectral slices with a smaller uniform bandwidth. Still, the signals generated and measured using the setup in Fig. 4 already show excellent signal quality, see Figs. 5b and 6. To the best of our knowledge, the 320 GBd demonstrated in our transmission experiments represent the highest symbol rate so far achieved for “fully coherent” QAM data signals that do not rely on optical time-division multiplexing (OTDM)-based schemes, for which subsequent symbols do not have a fixed phase relationship and where the pulse shape is defined by a mode-locked laser rather than synthesized digitally^55^.

### Application potential

We believe that our demonstration of high-bandwidth OAWG and OAWM can unlock a range of highly interesting applications, e.g., in the context of broadband test and measurement instrumentation. This could hit an increasing demand in the optical telecommunication industry: While the analog bandwidth of optical transmitters and receivers could be steadily increased over recent years by exploiting advanced complementary metal-oxide semiconductor (CMOS) nodes^56–60^, it became increasingly difficult for traditional test and measurement instruments such as real-time oscilloscopes and arbitrary waveform generators (AWGs) to keep pace with the evolution of optical transceivers in terms of bandwidth and signal quality. More specifically, the most broadband commercially available AWG (Keysight M8199B) offers a bandwidth of only 80 GHz, while the most broadband transceiver modules already achieve symbol rates of up to 200 GBd^60^, which requires a Nyquist bandwidth of at least 100 GHz. The key obstacle towards further increasing the performance of advanced test and measurement equipment is the fact that these systems can usually not leverage advanced CMOS technology nodes due to commercial aspects, that apply in addition to technological challenges: The mask design for a single 3 nm CMOS chip requires typical R&D investments of the order of $40 M^61^, which can be justified for large-volume markets such as optical transceivers, but which are unrealistic for medium- or low-volume applications such as test and measurement equipment. In this context, optoelectronic signal processing as proposed and demonstrated in our work can be an attractive solution, allowing to push the bandwidth of optical signal generation and detection far beyond the limitations of current all-electronic systems while maintaining superior signal quality, see Fig. 5. We hence believe that OAWG- and OAWM-based test and measurement systems could serve as tools for exploring the potential of ultra-high-symbol-rate communications on a proof-of-concept level and for characterizing and optimizing future transceiver technologies and transmission systems. Interesting questions that might be addressed by such OAWG and OAWM equipment are related to additional penalties that are associated with high symbol rates in long-distance links, comprising, e.g., non-linear interference noise (NLIN), equalization-enhanced phase noise (EEPN), and increased complexity of digital dispersion compensation^62–64^. Moreover, OAWG- and OAWM-based testbeds could allow for experimental studies of optimized DSP schemes for high bandwidth transceivers, including, e.g., the possibility to use digital subcarrier multiplexing^63,64^.

Beyond the generation of optical waveforms, we believe that our schemes can unlock new applications in the field of microwave and terahertz photonics. Examples comprise photonic-electronic waveform-generation schemes^65^ and terahertz communications^66,67^, both of which rely on combining the OAWG transmitter with a high-speed photodetector that converts the optical waveform back to the electronic domain.

One might also consider the question to which extent OAWG and OAWM techniques might be used in real optical communication systems. In this context, it should be noted that high symbol rates in optical communications are not valuable per se, unless they allow to increase the overall efficiency of the underlying link, e.g., by reducing the number of components such as lasers, modulators, photodetectors, ADCs, DACs, or DSP engines, that are required to cover a certain optical bandwidth. This is a delicate balance, in which the bandwidth of electronic and optoelectronic devices, as well as additional effects like the above-mentioned NLIN, EEPN, and increased complexity of digital dispersion compensation play an important role. Boosting the symbol rate through OAWG- and OAWM-based transceiver schemes as demonstrated in our experiments would incur all these challenges, without providing the benefit of requiring less components—each of the four OAWG channels has approximately the same hardware complexity as an independent coherent transmitter, not to talk of the additional effort of phase stabilization. Hence, the application of OAWG and OAWM-based schemes in optical communication systems will only become of interest if additional advantages, such as highly dynamic fully software-defined usage of spectral resources^68^, or the compensation of nonlinear effects that rely on the acquisition of wide optical bandwidths^69^ can be leveraged. Clearly, such applications would require fully integrated OAWG and OAWM engines that combine robustness with the amenability to low-cost mass production.

### Summary

We have demonstrated the generation and transmission of fully coherent 16QAM, 32QAM, and 64QAM signals at record-high symbol rates up to 320 GBd by combining comb-based OAWG and OAWM. To the best of our knowledge, our work represents the first OAWG demonstration using actively phase-stabilized spectral slices, leading to targeted optical waveform synthesis at the highest bandwidth so far achieved in any OAWG experiment. By multiplexing parallel ADC and DAC arrays in the optical domain, our concept renders the bandwidth of the optical signal independent of the bandwidth of individual electronic interfaces. This may offer a path forward towards ultra-broadband photonic-electronic signal generators that are relevant in a variety of technical and scientific applications.

## Materials and methods

A detailed description of the materials and methods is provided in the attached supplementary document (Supplementary information 1). In Section S1 of Supplementary information 1, the generation of the error signal for the feedback-controlled phase stabilization is discussed in detail. The system calibration techniques are explained in Section S2, where we also estimate the signal-to-noise-and-distortion ratio (SNDR) achieved by the OAWG system. Section S3 provides a definition of the constellation signal-to-noise ratio (CSNR), which is used in Figs. 5b and 6a to quantify the signal quality. In Section S5, we describe the methodology and the experimental setup used for measuring 320 GBd QAM signals at various optical signal-to-noise ratio (OSNR) levels.

## Supplementary information

Supplementary Information for Optical Arbitrary Waveform Generation (OAWG) Using Actively Phase-Stabilized Spectral Stitching

## Data Availability

Data underlying the results presented in this paper may be obtained from the authors upon reasonable request.
