# Supplementary material for: Optical arbitrary waveform generation (OAWG) using actively phase-stabilized spectral stitching
Source: Light Sci Appl. 2025 Sep 29;14:353. doi: 10.1038/s41377-025-01937-4 (PMC12479824; doi:10.1038/s41377-025-01937-4)
Supplement: Supplementary file 1 — Supplementary Information for Optical Arbitrary Waveform Generation (OAWG) Using Actively Phase-Stabilized Spectral Stitching [file 41377_2025_1937_MOESM1_ESM.pdf]

*Supplementary Information for*

# Optical Arbitrary Waveform Generation (OAWG) Using Actively Phase-Stabilized Spectral Stitching


Daniel Drayss[1,2,3,+,*], Dengyang Fang[1,3,+], Alban Sherifaj[1,+], Huanfa Peng[1], Christoph Füllner[1], Thomas Henauer[4], Grigory Lihachev[5], Lennart Schmitz[1], Tobias Harter[1], Wolfgang Freude[1], Sebastian Randel[1], Tobias J. Kippenberg[5,6], Thomas Zwick[4], and Christian Koos[1,2,3,6,**]

[1]*Institute of Photonics and Quantum Electronics (IPQ), Karlsruhe Institute of Technology (KIT), 76131 Karlsruhe, Germany*
[2]*Institute of Microstructure Technology (IMT), Karlsruhe Institute of Technology (KIT), 76344 Eggenstein-Leopoldshafen, Germany*
[3]*Teragear GmbH, 76227 Karlsruhe, Germany*
[4]*Institute of Radio Frequency Engineering and Electronics (IHE), Karlsruhe Institute of Technology (KIT), 76131 Karlsruhe, Germany*
[5]*Institute of Physics, Swiss Federal Institute of Technology Lausanne (EPFL), 1015 Lausanne, Switzerland*
[6]*Deeplight SA, 1025 St Sulpice, Switzerland*
[+]*Contributed equally.* [*]*daniel.drayss@kit.edu.* [**]*christian.koos@kit.edu*


This document provides supplementary information to the research paper entitled "Optical Arbitrary Waveform Generation (OAWG) Using Actively Phase-Stabilized Spectral Stitching:". In Section S1, we discuss the generation of the error signal for the feedback-controlled phase stabilization. The system calibration techniques are explained in Section S2, where we also estimate the signal-to-noise-and-distortion ratio (SNDR) achieved by the OAWG system. Section S3 provides a definition of the constellation signal-to-noise ratio (CSNR), which is used in the main manuscript to quantify the signal quality. Section S4 describes the methodology and the experimental setup used for measuring 320 GBd QAM signals at various optical signal-to-noise ratio (OSNR) levels.

## S1. Mathematical derivation of error-signal generation for closed loop phase control

In the main manuscript, we stated that the phase error $\Delta\varphi(t)$ between two overlapping tributary signals, e.g., $\underline{a}_{\text{in},1}(t)$ and $\underline{a}_{\text{in},2}(t)\exp\left(\text{j}\Delta\varphi(t)\right)$ as in Fig. S1 (a) below, can be measured by using a suitable passive combiner in conjunction with a low-speed balanced photodetector (BPD). In the following, we provide a more detailed derivation of the underlying mathematical relations for either a 120° optical hybrid or a 90° optical hybrid as a passive combining element. For the experiments discussed in the main manuscript, we have used a 90° optical hybrid, which is a standard and readily available component in coherent optical communications. The digital proportional-integral (PI) controller was implemented on an open-source software measurement and control board (Red Pitaya STEMlab 125 with Xilinx Zynq 7010) and a piezo-based fiber stretcher (FPS-003, General Photonics now part of Luna Innovations) with a tuning range of $55\pi$ was used as a phase shifter (PS).

### S1.1. Error signal generation using 120°optical hybrid

Figure S1 (a) shows a signal-combining element (SCE) using a 120°optical hybrid as a passive combiner, which may, e.g., be implemented based on a 3×3 multi-mode interference (MMI) coupler. All output signals and components related to the generation of the error signal $U_{\text{err}}(t)$ are marked in green. For simplicity, the signal tributaries $\underline{a}_{\text{in},1}(t)$ and $\underline{a}_{\text{in},2}(t)$ are assumed to spectrally overlap and to contain identical signal components within the overlap region (OR), $\left[f_2 - B, f_1 + B\right]$, see gray area in Fig. S1 (b). The signal components of the two tributaries in the overlap region are referred to by $\underline{a}_{\text{OR}}(t)$, where the corresponding spectrum $\underline{\tilde{a}}_{\text{OR}}(f)$ is given by

$$\underline{\tilde{a}}_{\text{OR}}(f) = \begin{cases} \underline{\tilde{a}}_{\text{in},1}(f) = \underline{\tilde{a}}_{\text{in},2}(f) & \text{for } f \in \left[f_2 - B, f_1 + B\right] \\ 0 & \text{otherwise.} \end{cases} \tag{S1}$$

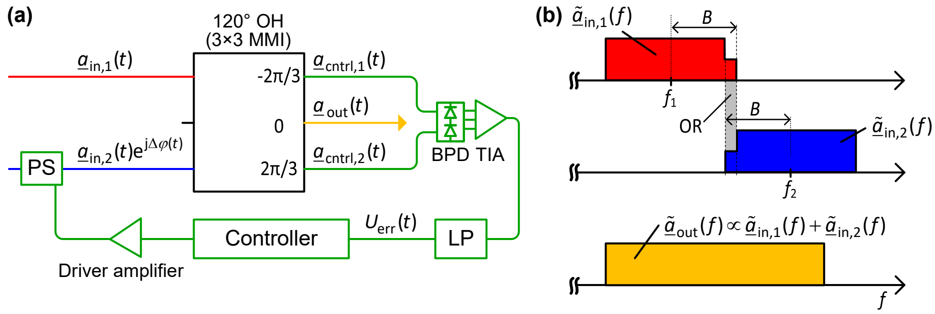


**Fig. S1.** Actively phase-stabilized combining of two overlapping spectral slices using a signal-combining element (SCE) based on a 120° optical hybrid (OH). **(a)** Conceptual setup of an SCE using a 3x3 multi-mode interference (MMI) coupler, for which the center input port remains unused. The MMI coupler superimposes the input signals $\underline{a}_{in,1}(t)$ and $\underline{a}_{in,2}(t)\exp(j\Delta\varphi(t))$, having a phase error $\Delta\varphi(t)$ with relative phases of $-2\pi/3$, 0, and $2\pi/3$ at the various output ports. The outer output ports (green) are used to generate the optical control signals $\underline{a}_{cntrl,1}(t)$ and $\underline{a}_{ctnrl,2}(t)$, whereas the combined output signal $\underline{a}_{out}(t)$ is generated at the center "zero phase" port ("0", yellow). The electrical error signal $U_{err}(t)$ is obtained by feeding the two optical control signals $\underline{a}_{cntrl,1}(t)$ and $\underline{a}_{ctnrl,2}(t)$ to a low-speed balanced photodetector (BPD), which is followed by a transimpedance amplifier (TIA) and a low-pass filter (LP). In our model, the LP represents the bandwidth limitations of the entire loop, including the BPD, the TIA, the controller, the driver amplifier, and the phase shifter (PS). The low-pass filtered error signal $U_{err}(t)$ is essentially proportional to phase error $\Delta\varphi(t)$, using a linear approximation close to the desired operating point $\Delta\varphi = 0$, see Eq. S10. A proportional-integral (PI) controller can hence be used to drive the phase shifter (PS) and to compensate for the measured phase error $\Delta\varphi(t)$. **(b)** Spectra $\tilde{\underline{a}}_{in,1}(f)$ and $\tilde{\underline{a}}_{in,2}(f)$ of the two tributaries $\underline{a}_{in,1}(t)$ and $\underline{a}_{in,2}(t)$ along with the spectrum of the targeted output waveform $\underline{a}_{out}(t) \propto \underline{a}_{in,1}(t) + \underline{a}_{in,2}(t)$ for $\Delta\varphi(t) = 0$. The spectra of the two tributaries are overlapping within the overlap region (OR, $f \in [f_2 - B, f_1 + B]$, gray).

To derive a mathematical model of the SCE, we start from the scattering matrix of an ideal 3×3 MMI [1], where the center input port is unused, Fig. S1 (a),

$$\begin{bmatrix} \underline{a}_{cntrl,1}(t) \\ \underline{a}_{out}(t) \\ \underline{a}_{cntrl,2}(t) \end{bmatrix} = \frac{1}{\sqrt{3}} \begin{bmatrix} -1 & e^{j\pi/3} \\ 1 & 1 \\ e^{j\pi/3} & -1 \end{bmatrix} \begin{bmatrix} \underline{a}_{in,1}(t) \\ \underline{a}_{in,2}(t)e^{j\Delta\varphi(t)} \end{bmatrix}. \tag{S2}$$

The output signal $\underline{a}_{out}(t)$ of the SCE is tapped at the center output port, labeled by a relative phase of "0" in Fig. S1 (a) and thus contains the sum of the two optical input signals $\underline{a}_{in,1}(t)$ and $\underline{a}_{in,2}(t)e^{j\Delta\varphi(t)}$,

$$\underline{a}_{out}(t) = \frac{1}{\sqrt{3}} \left[\underline{a}_{in,1}(t) + \underline{a}_{in,2}(t)e^{j\Delta\varphi(t)}\right]. \tag{S3}$$

To obtain the targeted output signal, the slowly varying phase error $\Delta\varphi(t)$ between the two tributaries at the input of the MMI must be kept at zero by the feedback loop and the phase shifter (PS). The error signal required for the control loop is generated by balanced detection of the two optical signals $\underline{a}_{cntrl,1}(t)$ and $\underline{a}_{cntrl,2}(t)$, which are obtained at the upper and the lower MMI output labeled by relative phases $-2\pi/3$ and $2\pi/3$ in Fig. S1 (a) and by subsequent low-pass filtering,

$$U_{err}(t) = H_{LP}(t) * \left[\left|\underline{a}_{cntrl,1}(t)\right|^2 - \left|\underline{a}_{cntrl,2}(t)\right|^2\right]. \tag{S4}$$

In this relation, $H_{LP}(t)$ is the impulse response of an equivalent low-pass filter, which represents the bandwidth limitations of the entire loop, including not only the BPD and the transimpedance amplifier (TIA) but also the lowpass characteristics of the subsequent components such as the controller, the driver amplifier and the phase shifter, Fig. S1 (a). The bandwidth $B_{LP}$ of the lowpass filter is chosen big enough to enable tracking of phase fluctuations on a millisecond time scale, but not too large as to suppress noise in the feedback loop. In our experiment, a bandwidth $B_{LP} \approx 10\,\text{kHz}$ turned out to be a good choice. Inserting Eq. S2 into Eq. S4

leads to

$$\begin{aligned} U_{\mathrm{err}}(t) &= H_{\mathrm{LP}}(t) * \left[ -\frac{4}{3} \sin\left(2\pi/3\right) \Im\left\{ \underline{a}_{\mathrm{in},1}(t) \underline{a}^*_{\mathrm{in},2}(t) \mathrm{e}^{-\mathrm{j}\Delta\varphi(t)} \right\} \right] \\ &= H_{\mathrm{LP}}(t) * \left[ -\frac{2}{\sqrt{3}} \Im\left\{ \underline{a}_{\mathrm{in},1}(t) \underline{a}^*_{\mathrm{in},2}(t) \mathrm{e}^{-\mathrm{j}\Delta\varphi(t)} \right\} \right], \end{aligned} \tag{S5}$$

where $\Im\{\cdot\}$ refers to the imaginary part of the respective complex number. Assuming that the bandwidth $B_{\mathrm{LP}}$ of the lowpass filter is sufficiently broad to capture the phase drift $\Delta\varphi(t)$ of our setup without distortions, i.e., $\exp\{-\mathrm{j}\Delta\varphi(t)\}$ is approximately constant within the duration of the low-pass impulse response $H_{\mathrm{LP}}(t)$, we can simplify Eq. S5 by moving the phase-error term $\exp\{-\mathrm{j}\Delta\varphi(t)\}$ out of the convolution integral,

$$\begin{aligned} U_{\mathrm{err}}(t) &\propto \Im\left\{ H_{\mathrm{LP}}(t) * \left[ \underline{a}_{\mathrm{in},1}(t) \underline{a}^*_{\mathrm{in},2}(t) \mathrm{e}^{-\mathrm{j}\Delta\varphi(t)} \right] \right\} \\ &= \Im\left\{ \int_{-\infty}^{\infty} H_{\mathrm{LP}}(\tau) \underline{a}_{\mathrm{in},1}(t-\tau) \underline{a}^*_{\mathrm{in},2}(t-\tau) \mathrm{e}^{-\mathrm{j}\Delta\varphi(t-\tau)} \mathrm{d}\tau \right\} \\ &\approx \Im\left\{ \mathrm{e}^{-\mathrm{j}\Delta\varphi(t)} \int_{-\infty}^{\infty} H_{\mathrm{LP}}(\tau) \underline{a}_{\mathrm{in},1}(t-\tau) \underline{a}^*_{\mathrm{in},2}(t-\tau) \mathrm{d}\tau \right\} \\ &= \Im\left\{ \mathrm{e}^{-\mathrm{j}\Delta\varphi(t)} U_0(t) \right\}, \end{aligned} \tag{S6}$$

In this relation, the control-voltage amplitude $U_0(t)$ corresponds to the low-pass-filtered interference of the phase-stabilized overlapping signal components $\underline{a}_{\mathrm{in},1}(t)$ and $\underline{a}_{\mathrm{in},2}(t)$ , while the phase error is contained in the pre-factor $\exp\{-\mathrm{j}\Delta\varphi(t)\}$.

$$U_0(t) = \int_{-\infty}^{\infty} H_{\mathrm{LP}}(t-\tau) \underline{a}_{\mathrm{in},1}(\tau) \underline{a}^*_{\mathrm{in},2}(\tau) \mathrm{d}\tau. \tag{S7}$$

Note that the low-pass filter bandwidth $B_{\mathrm{LP}}$ is much smaller than the bandwidth $B_{\mathrm{OR}}$ of the overlap region $B_{\mathrm{OR}}$, $B_{\mathrm{OR}}/B_{\mathrm{LP}} > 10^5$ in case of our experiments. As a consequence, for a given spectral component $\underline{\tilde{a}}_{\mathrm{in},1}(f_{\mathrm{a}})$ of the first tributary, the control-voltage amplitude $U_0(t)$ only contains beat signals with directly adjacent spectral components $\underline{\tilde{a}}_{\mathrm{in},2}(f_{\mathrm{b}})$, $|f_{\mathrm{a}} - f_{\mathrm{b}}| < B_{\mathrm{LP}}$, of the second tributary. The contribution of $\underline{\tilde{a}}_{\mathrm{in},1}(f)$ and $\underline{\tilde{a}}_{\mathrm{in},2}(f)$ to the control-voltage amplitude $U_0(t)$ in Eq. S7 is hence essentially limited to spectral regions, in which both $\underline{\tilde{a}}_{\mathrm{in},1}(f)$ and $\underline{\tilde{a}}_{\mathrm{in},2}(f)$ are nonzero, i.e., the overlap region $f \in \mathrm{OR}$. The tributaries $\underline{a}_{\mathrm{in},1}(t)$ and $\underline{a}_{\mathrm{in},2}(t)$ in Eq. S7 can thus be replaced by the common signal component $\underline{a}_{\mathrm{OR}}(t)$ within the overlap region, Eq. S1,

$$U_0(t) \approx H_{\mathrm{LP}}(t) * |\underline{a}_{\mathrm{OR}}(t)|^2, \qquad \text{for } B_{\mathrm{LP}} \ll B_{\mathrm{OR}}. \tag{S8}$$

We further make use of the fact that communication signals as used in our experiments are stationary and that both the symbol rate and the bandwidth of the overlap region greatly exceed the bandwidth $B_{\mathrm{LP}}$ of the low-pass filter. The low-pass filter hence averages over many symbols and we can assume that the resulting mean power $P_{\mathrm{OR}}$ of the signal components in the overlap region is constant, $P_{\mathrm{OR}} \propto H_{\mathrm{LP}}(t) * |\underline{a}_{\mathrm{OR}}(t)|^2 \approx \mathrm{const.}$ The error signal in Eq. S6 can thus be re-written by replacing $U_0(t)$ by a constant $U_0$,

$$U_{\mathrm{err}}(t) \propto U_0 \Im\left\{ \mathrm{e}^{-\mathrm{j}\Delta\varphi(t)} \right\} = U_0 \sin\left\{\Delta\varphi(t)\right\}. \tag{S9}$$

Once the feedback-based phase control is activate, the residual phase error $\Delta\varphi(t)$ is small, $\Delta\varphi(t) \ll 1$. We can thus linearize Eq. S9 about the desired operating point $\Delta\varphi = 0$ of the control loop,

$$U_{\mathrm{err}}(t) \propto \sin\left\{\Delta\varphi(t)\right\} \approx \Delta\varphi(t), \qquad \text{for } |\Delta\varphi| \ll 1. \tag{S10}$$

The error signal $U_{\mathrm{err}}(t)$ is hence approximately proportional to the phase error $\Delta\varphi(t)$, see Eq. 6 in the main manuscript, and can be used to implement a linear PI control loop to stabilize the optical phase as demonstrated in Fig. 3 of the manuscript. Additional reset mechanisms may need to be implemented to cope with the limited range of the phase shifter in case of fiber-based setups, where the phase drift can exceed the phase-shifter range.

Note that for arbitrary waveforms, the condition of constant average power in the overlap regions may be violated, i.e., $U_0(t) \neq \mathrm{const.}$, prohibiting the generation of clean error signals. Likewise, there exist waveforms

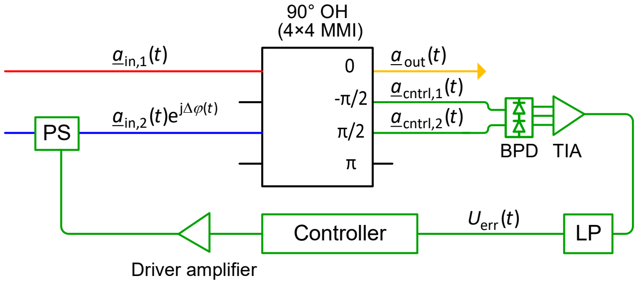


**Fig. S2.** Concept for actively-phase stabilized combining of two overlapping spectral slices using a signal-combining element (SCE) based on a 90° optical hybrid (OH), which may be implemented via a 4×4 multi-mode interference (MMI) coupler. The 2nd and 4th input ports as well as the 4th output port remain unused. The MMI superimposes the input signals with relative phases of 0, $-\pi/2$, and $\pi/2$ at the various output ports. The two overlapping spectral slices $\underline{a}_{in,1}(t)$ and $\underline{a}_{in,2}(t)\exp(j\Delta\varphi(t))$ with phase error $\Delta\varphi(t)$ are combined at the "zero-phase" port ("0"), leading to the output signal $\underline{a}_{out}(t)$. The error signal $U_{err}(t)$ is generated by feeding the output signals $\underline{a}_{cntrl,1}(t)$ ("$-\pi/2$") and $\underline{a}_{ctnrl,2}(t)$ ("$\pi/2$") to a low-speed balanced photodetector (BPD) followed by a transimpedance amplifier (TIA). Upon low-pass (LP) filtering, the resulting error signal $U_{err}(t)$ is essentially proportional to the phase error $\Delta\varphi(t)$ for a linear approximation close to the desired operating point $\Delta\varphi = 0$. A proportional-integral (PI) controller is used to drive a phase shifter (PS) that compensates for the measured phase error $\Delta\varphi(t)$.

that do not contain spectral components inside the overlap region, resulting in no error signal being generated at all. To overcome these difficulties and generate valid feedback signals for arbitrary waveforms, we may rely on well-defined pilot tones inside the spectral overlap regions instead. By designing these pilot tones to destructively interfere at the main output of the MMI, additional distortions of this approach can be avoided.

### S1.2. Error signal generation using 90°optical hybrid

The generation of the error signal $U_{err}(t)$ for the active phase control based on a 90°optical hybrid as a passive combiner, e.g., implemented as 4×4 MMI, is almost identical to the generation based on a 120°optical hybrid as discussed in the previous section. The setup of the SCE is depicted in Fig. S2.

In analogy to Eq. S2, the three output signals $\underline{a}_{out}(t)$, $\underline{a}_{cntrl,1}(t)$, and $\underline{a}_{cntrl,2}(t)$ at the output ports 1,2,and 3 of an ideal 4×4 MMI can be calculated from the input signals $\underline{a}_{in,1}(t)$ and $\underline{a}_{in,2}(t)\exp\{j\Delta\varphi(t)\}$ connected to the input ports 1 and 3, respectively [1],

$$\begin{bmatrix} \underline{a}_{out}(t) \\ \underline{a}_{cntrl,1}(t) \\ \underline{a}_{cntrl,2}(t) \end{bmatrix} = \frac{1}{2} \begin{bmatrix} 1 & 1 \\ 1 & -j \\ 1 & j \end{bmatrix} \begin{bmatrix} \underline{a}_{in,1}(t) \\ \underline{a}_{in,2}(t)e^{j\Delta\varphi(t)} \end{bmatrix}. \tag{S11}$$

Using Eq. S11, the targeted output signal $\underline{a}_{out}(t)$ as well as the control signal $U_{err}(t)$ are again calculated in analogy to Eq. S3 and Eq. S4, yielding

$$U_{err}(t) \approx H_{LP}(t) * \left[-\Im\left\{\underline{a}_{in,1}(t)\underline{a}^{*}_{in,2}(t)e^{-j\Delta\varphi(t)}\right\}\right]. \tag{S12}$$

We use the same assumptions as in the previous section, i.e., that the low-pass filter bandwidth $B_{LP}$ is, on the one hand, sufficiently large to not distort the phase error $\Delta\varphi(t)$ and, on the other hand, still much smaller than the bandwidth $B_{OR}$ of the overlap region, $B_{LP} \ll B_{OR}$ such that noise and fast modulations of the input signals are suppressed. For stationary signals, we find in accordance to Eq. S10 that $U_{err}(t) \propto \Delta\varphi(t)$ close to the desired operating point $\Delta\varphi = 0$.

## S2. Calibration

### S2.1. Electro-optic calibration of OAWG transmitter

To compensate linear distortions introduced by the various system components, we pre-distort the drive signals $I_\nu(t)$ and $Q_\nu(t)$ of the various in-phase and quadrature modulators (IQMs), which requires precise

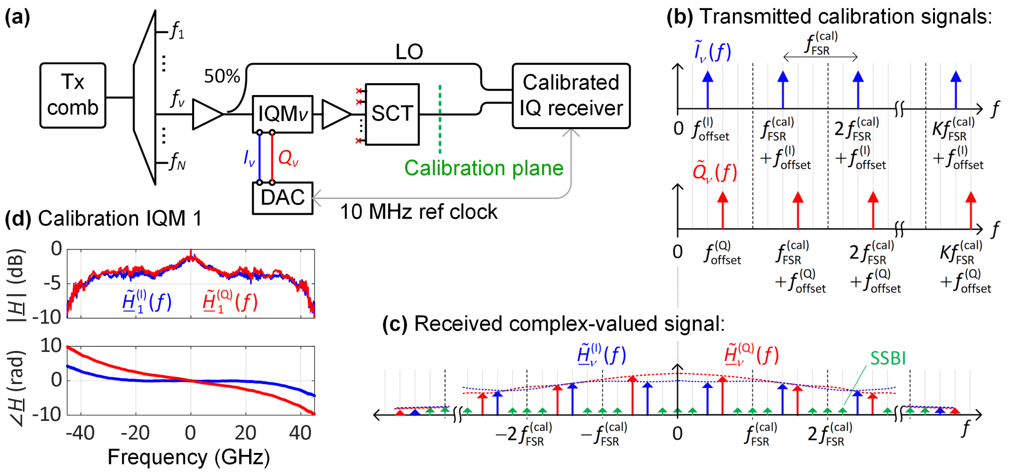


**Fig. S3.** Concept and experimental setup for electro-optic calibration of the OAWG transmitter. **(a)** Experimental setup for calibration of the $\nu$-th transmitter channel. The $\nu$-th comb line at frequency $f_\nu$ of the transmitter comb (Tx comb) is amplified and split equally into a local oscillator (LO) tone, that is fed to a calibrated IQ receiver (IQR), and an optical carrier, that is fed to the IQ modulator (IQM$\nu$) associated with the channel of interest. The in-phase and quadrature inputs of the IQM are driven by the associated radio frequency (RF) calibration signals $I_\nu(t)$ and $Q_\nu(t)$, both of which are designed to exhibit comb-like power spectra, see Subfigure (b). The optical output signal of IQM$\nu$ is amplified, sent through the signal-combining tree (SCT), and received by the calibrated IQR. **(b)** Visualization of the spectra $\tilde{I}_\nu(f)$ (blue) and $\tilde{Q}_\nu(f)$ (red) of the RF calibration signals generated by the digital-to-analog converters (DACs). Note that the two combs are spectrally interleaved to ease the separation of the in-phase and quadrature signal components at the IQR. **(c)** Spectrum of the complex-valued signal received by the calibrated IQR. The transmitter transfer functions $\underline{\tilde{H}}_\nu^{(\mathrm{I})}(f)$ and $\underline{\tilde{H}}_\nu^{(\mathrm{Q})}(f)$ can be extracted by determining the amplitude and phase of the various comb tones and by interpolating for frequencies in between. The spectral position of the calibration comb lines is engineered to avoid distortions by signal-signal beat interference (SSBI, green). **(d)** Amplitude and phase of exemplary transfer functions $\underline{\tilde{H}}_\nu^{(\mathrm{I})}(f)$ and $\underline{\tilde{H}}_\nu^{(\mathrm{Q})}(f)$ for transmitter $\nu = 1$.

knowledge of the associated frequency-dependent transfer functions $\underline{\tilde{H}}_\nu^{(\mathrm{I})}(f)$ and $\underline{\tilde{H}}_\nu^{(\mathrm{Q})}(f)$, $\nu = 1, \ldots, N$, see Eq. 5 in the main manuscript. To this end, we characterize each transmitter channel in a dedicated measurement by means of an IQ receiver (IQR) that was calibrated beforehand using an ultra-stable femtosecond laser (Menhir 1550), see [2–4] for details. The experimental setup for the calibration of the $\nu$-th IQM is shown in Fig. S3 (a). The $\nu$-th comb line at frequency $f_\nu$ is amplified and split into two copies, where one copy is used as an optical carrier and fed to IQM$\nu$ and the other copy is used as local oscillator (LO) tone for homodyne reception. The optical carrier fed to IQM$\nu$ is modulated by the calibration signals $I_\nu(t)$ and $Q_\nu(t)$, and the generated optical waveform is subsequently amplified, propagates though the signal-combining tree (SCT), see Fig. 1 in the main manuscript, and is detected by the calibrated IQR. Note that we use a homodyne detection setup and synchronize the analog-to-digital converters (ADCs) of the calibrated IQR to the digital-to-analog converters (DACs) that drive the IQMs to minimize the impact of optical phase noise at the receiver and to eliminate any frequency offset between the optical carrier and the LO. Because the calibration must include the time delay between the in-phase and quadrature components (IQ-skew) we measure both transfer functions, $\underline{\tilde{H}}_\nu^{(\mathrm{I})}(f)$ and $\underline{\tilde{H}}_\nu^{(\mathrm{Q})}(f)$ simultaneously. To this end, we generate two interleaved radio frequency (RF) frequency combs as calibration signals, where one RF comb is use as a drive signal $I_\nu(t)$ for the in-phase component of IQM$\nu$ whereas the other RF comb serves as a drive signal $Q_\nu(t)$ for the quadrature component. The two combs have the same free spectral range (FSR) $f_{\mathrm{FSR}}^{(\mathrm{cal})}$ but different frequency offsets $f_{\mathrm{offset}}^{(\mathrm{I})}$ and $f_{\mathrm{offset}}^{(\mathrm{Q})}$,

see Fig. S3 (b),

$$I_\nu(t) = \sum_{k=0}^{K} \cos\left[2\pi\left(kf_{\mathrm{FSR}}^{(\mathrm{cal})} + f_{\mathrm{offset}}^{(\mathrm{I})}\right)t + \varphi_k^{(\mathrm{I})}\right], \tag{S13}$$

$$Q_\nu(t) = \sum_{k=0}^{K} \cos\left[2\pi\left(kf_{\mathrm{FSR}}^{(\mathrm{cal})} + f_{\mathrm{offset}}^{(\mathrm{Q})}\right)t + \varphi_k^{(\mathrm{Q})}\right]. \tag{S14}$$

In this relation, $K$ is the total number of comb lines of the calibration signal and $\varphi_k^{(\mathrm{I})}$ and $\varphi_k^{(\mathrm{Q})}$ are the initial phases of the various comb lines. The distinct frequency offsets $f_{\mathrm{offset}}^{(\mathrm{I})}$ and $f_{\mathrm{offset}}^{(\mathrm{Q})}$ allow to easily separate the components related to the in-phase and quadrature drive signals at the output of the calibrated IQR, Fig. S3 (c). For each comb-tone frequency, we can directly extract the complex-valued transfer functions $\underline{\tilde{H}}_\nu^{(\mathrm{I})}(f)$ and $\underline{\tilde{H}}_\nu^{(\mathrm{Q})}(f)$ from the amplitude and phase of the received comb lines, indicated in red and blue in Fig. S3 (c), and these data points can then be used to estimate $\underline{\tilde{H}}_\nu^{(\mathrm{I})}(f)$ and $\underline{\tilde{H}}_\nu^{(\mathrm{Q})}(f)$ at any other frequency by interpolation. Exemplary amplitude and phase transfer functions as measured for IQM1 are shown in Fig. S3 (d).

Note that the accuracy of the obtained transfer functions $\underline{\tilde{H}}_\nu^{(\mathrm{I})}(f)$ and $\underline{\tilde{H}}_\nu^{(\mathrm{Q})}(f)$ may be impaired by amplified spontaneous emission (ASE) noise, ADC and DAC noise, signal-signal beat interference (SSBI), and by the accuracy of the IQR calibration. In the following, we will briefly discuss the different impairments and possible mitigation measures:

- **ASE noise:** Amplified spontaneous emission (ASE) noise cannot be fully avoided. However, its effect on the calibration result can be reduced by averaging multiple calibration measurements.

- **ADC and DAC noise:** The root-mean-square (RMS) voltage noise added by an ADC or DAC usually takes a fixed percentage of the full-scale voltage, and the signal is typically adjusted to fill the full input or output range of the ADC or DAC, respectively. As a result, the achievable signal-to-noise ratio (SNR) depends on the peak-to-average power ratio (PAPR) of the signal. To reduce the PAPR and consequently increase the SNR of the transmitted and received signals, the initial phases $\varphi_k^{(\mathrm{I})}$ and $\varphi_k^{(\mathrm{Q})}$ are chosen such that the comb spectra $\tilde{I}_\nu(f)$ and $\tilde{Q}_\nu(f)$ have a quadratic phase profile. This is equivalent to chirping the time domain pulse trains $I_\nu(t)$ and $Q_\nu(t)$ such that the gaps between the periodic pulses vanish and the optical power of the calibration signal is approximately constant. Additionally, ADC and DAC noise can be further reduced by averaging multiple calibration measurements.

- **Signal-signal beat interference (SSBI):** The common-mode rejection ratio (CMRR) of BPDs is generally limited, which results in SSBI distorting the received waveforms and leading to deterministic errors in the retrieved transfer functions $\underline{\tilde{H}}_\nu^{(\mathrm{I})}(f)$ and $\underline{\tilde{H}}_\nu^{(\mathrm{Q})}(f)$. These impairments are systematic and cannot be removed by averaging more measurements. However, a proper choice of the frequency offsets $f_{\mathrm{offset}}^{(\mathrm{I})}$ and $f_{\mathrm{offset}}^{(\mathrm{Q})}$ Fig. S3 (b), allows eliminate the effect of SSBI entirely. For example, by choosing $f_{\mathrm{offset}}^{(\mathrm{I})} = 0.4 \times f_{\mathrm{FSR}}^{(\mathrm{cal})}$ and $f_{\mathrm{offset}}^{(\mathrm{Q})} = 0.6 \times f_{\mathrm{FSR}}^{(\mathrm{cal})}$ as in Fig. S3 (b), the SSBI spectrum at the receiver will only contain beat notes at frequencies $f_{\mathrm{SSBI}} = kf_{\mathrm{FSR}}^{(\mathrm{cal})}$, and $f_{\mathrm{SSBI}} = (k \pm 0.2)f_{\mathrm{FSR}}^{(\mathrm{cal})}$ with integer $k$ (green comb lines in Fig. S3 (c)) and thus not overlap with the desired signal-LO beat notes at frequencies $(k+0.4)f_{\mathrm{FSR}}^{(\mathrm{cal})}$ (blue comb lines in Fig. S3 (c)) and $(k+0.6)f_{\mathrm{FSR}}^{(\mathrm{cal})}$ (red comb lines in Fig. S3 (c)), respectively.

- **Calibration errors of the IQR:** The IQM calibration will directly inherit errors from the IQR calibration. Here, we investigate the impact of our receiver calibration on the retrieved transmitter transfer functions $\underline{\tilde{H}}_\nu^{(\mathrm{I})}(f)$ and $\underline{\tilde{H}}_\nu^{(\mathrm{Q})}(f)$ by repeating the transmitter calibration using a different IQR (IQR2). We find that the obtained transfer functions $\underline{\tilde{H}}_{\nu,\mathrm{IQR1}}^{(\mathrm{I})}(f)$, $\underline{\tilde{H}}_{\nu,\mathrm{IQR2}}^{(\mathrm{I})}(f)$, and $\underline{\tilde{H}}_{\nu,\mathrm{IQR1}}^{(\mathrm{Q})}(f)$, $\underline{\tilde{H}}_{\nu,\mathrm{IQR2}}^{(\mathrm{Q})}(f)$ differ only slightly. We quantify the difference by the average relative error $J$,

$$J = \frac{\int_{-B}^{B} \left|\underline{\tilde{H}}_{\nu,\mathrm{IQR1}}^{(\mathrm{I})}(f) - \underline{\tilde{H}}_{\nu,\mathrm{IQR2}}^{(\mathrm{I})}(f)\right|^2 \mathrm{d}f}{\int_{-B}^{B} \left|\underline{\tilde{H}}_{\nu,\mathrm{IQR1}}^{(\mathrm{I})}(f) + \underline{\tilde{H}}_{\nu,\mathrm{IQR2}}^{(\mathrm{I})}(f)\right|^2 \mathrm{d}f}, \tag{S15}$$

which for our transmitter calibrations is of the order of $-35\,\mathrm{dB}$ to $-45\,\mathrm{dB}$ for a bandwidth $B = 45\,\mathrm{GHz}$. Note, however, that both IQRs were calibrated using the same optical reference waveform (ORW), such that systematic errors originating from the ORW or receiver calibration procedure cannot be detected.

- **System nonlinearities:** System nonlinearities cannot be compensated using this approach, because the underlying system model is purely linear, see Eq. 5 in the main manuscript. Nevertheless, in some situations, e.g., when using semiconductor optical amplifiers (SOAs) with non-negligible pattern effects rather than more ideal erbium-doped fiber amplifiers (EDFAs), the calibration accuracy can be improved by choosing IQ calibration signals $I_\nu(t)$ and $Q_\nu(t)$ that have similar statistical properties as the IQ drive used for the target waveform. To this end, the phases $\varphi_k^{(\mathrm{I})}$ and $\varphi_k^{(\mathrm{Q})}$ of the various comb lines can be engineered. For example, choosing the phases $\varphi_k^{(\mathrm{I})}$ and $\varphi_k^{(\mathrm{Q})}$ randomly results in calibration signals $I_\nu(t)$ and $Q_\nu(t)$ with an approximately Gaussian amplitude distribution – similar to the IQ drive signals used for the generation of data signals in our experiments.

### S2.2. Calibration verification and SNR estimation

To validate the transmitter calibration and to measure the in-band noise, we generate 40 GBd 16QAM single-sideband signals using the various IQMs. The resulting optical power spectral density (PSD) $S(f)$ is recorded by a high-resolution optical spectrum analyzer (OSA, Waveanalyzer 1500s, Coherent Corp., Saxonburg, Pennsylvania, USA) at a resolution bandwidth (RBW) of RBW = 150 MHz, see Fig. S4. First, we observe that the generated single-sideband spectra are flat to the left of the respective carrier and that the positive sideband to the right of the respective carrier is well suppressed. This indicates a low level of remaining IQ imbalance and thus a good transmitter calibration and bias-point setting of the modulator. Next, we estimate the total in-band noise-and-distortion power level $P_{\mathrm{ND}}$ by integrating the optical PSD $S(f)$ within the nominally empty upper sidebands,

$$P_{\mathrm{ND}} = \sum_{\mu=1}^{4} \left( \int_{f_\mu+\delta}^{f_\mu+f_{\mathrm{FSR}}/2-\delta} S(f)\mathrm{d}f \right). \tag{S16}$$

In the above relation, $f_{\mathrm{FSR}} = 80\,\mathrm{GHz}$ is the spacing between the four optical carriers $f_\mu$, $\mu = 1, ..., 4$, and $\delta = 0.6\,\mathrm{GHz}$ is a small guard band that is used to avoid integrating into the roll-off regions of the spectrally adjacent root-raised cosine (RRC) signal spectra. Considering all four empty sidebands, the total bandwidth $B$ within which noise is integrated amounts to $B = 4\,(f_{\mathrm{FSR}}/2 - 2\delta) = 155.2\,\mathrm{GHz}$, see spectral regions marked in blue in Fig. S4. The associated average in-band noise PSD $S_{\mathrm{ND}} = P_{\mathrm{ND}}/B$ is indicated by a blue dashed line. The integrated frequency range includes ASE noise, spurs and noise from the DACs, noise and distortions from the electrical amplifiers driving the IQMs, as well as residual IQ crosstalk from the lower sidebands. The spurs marked with purple dashed arrows in Fig. S4, occur 30 GHz away from each optical carrier and originate from the DACs (Keysight M8194A). For the subsequent signal-to-noise-and-distortion ratio (SNDR) estimation we assume that the noise-and-distortion power $P_{\mathrm{ND}}$ is identical in both sidebands.

In a next step, we determine the total signal power $P_{\mathrm{S}}$ by integrating the PSD of the single-sideband signal $S(f)$ within the lower sideband and by subsequently subtracting the total noise-and-distortion power $P_{\mathrm{ND}}$,

$$P_{\mathrm{S}} = \sum_{\mu=1}^{4} \left( \int_{f_\mu-f_{\mathrm{FSR}}/2+\delta}^{f_\mu-\delta} S(f)\mathrm{d}f \right) - P_{\mathrm{ND}}. \tag{S17}$$

We again disregard the roll-off region of the RRC signal spectra for simplicity. The associated average signal PSD $S_{\mathrm{S}} = P_{\mathrm{S}}/B$ is indicated in Fig. S4 by a red dashed line. The SNDR $\mathrm{SNDR}_{\mathrm{Tx}}^{(155.2\,\mathrm{GHz})}$ of the four-channel single-sideband signal is then calculated by relating the total signal power $P_{\mathrm{S}}$ to the total noise-and-distortion power $P_{\mathrm{ND}}$,

$$\mathrm{SNDR}_{\mathrm{Tx}}^{(155.2\,\mathrm{GHz})} = 10\log_{10}\left(\frac{P_{\mathrm{S}}}{P_{\mathrm{ND}}}\right) = 25.3\,\mathrm{dB}. \tag{S18}$$

For comparison, we also determine the ASE-related SNR $\mathrm{SNR}_{\mathrm{ASE}}^{155.2\,\mathrm{GHz}}$, based on the out-of-band ASE noise. To this end, we calculate the average ASE noise PSD $S_{\mathrm{ASE}}$ by taking the mean of the noise PSD measured to

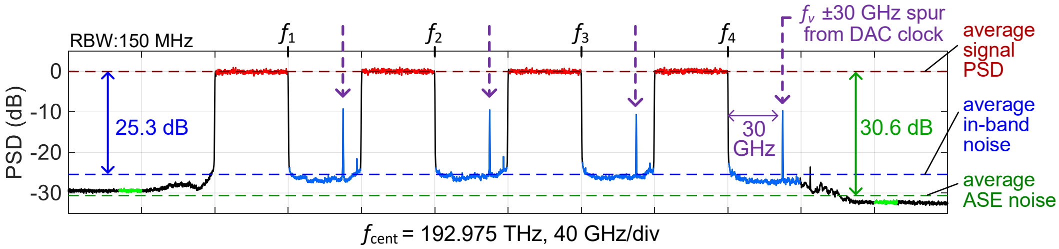


**Fig. S4.** Optical spectrum of a signal generated by the OAWG system and measured after passing through a linear polarizer. The measurement was taken using a high-resolution optical spectrum analyzer (Waveanalyzer 1500s, Coherent Corp., Saxonburg, Pennsylvania, USA, resolution bandwidth: 150 MHz). The spectrum consists of four identical single-sideband 40 GBd 16QAM signals occupying the low-frequency sideband of each transmitter channel. The configuration allows determining the single-polarization amplified spontaneous emission (ASE) noise (green dashed line) as well as the noise and distortions in the empty upper sidebands (blue dashed line), including ASE noise, spurs and noise from the digital-to-analog converters (DACs), noise from the electrical amplifiers driving the in-phase and quadrature modulators (IQMs), and IQ crosstalk from the lower sidebands. The spurs originating from the DACs (Keysight M8194A) occur ±30 GHz away from each optical carrier $f_1, ..., f_4$ and are marked with dashed purple arrows. Note that the spurious tones exist for both sidebands but can only be seen in the empty upper sideband.

the left $S_{\mathrm{ASE,l}}$ and to the right $S_{\mathrm{ASE,r}}$ of the signal, see green dashed line in Fig. S4,

$$S_{\mathrm{ASE}} = \frac{S_{\mathrm{ASE,l}} + S_{\mathrm{ASE,r}}}{2}. \tag{S19}$$

The ASE-related SNR $\mathrm{SNR}_{\mathrm{ASE}}^{155.2\,\mathrm{GHz}}$ is obtained by relating the total signal power $P_{\mathrm{S}}$ to the total ASE noise power within the bandwidth $B = 155.2\,\mathrm{GHz}$,

$$\mathrm{SNR}_{\mathrm{ASE}}^{(155.2\,\mathrm{GHz})} = 10\log_{10}\left(\frac{P_{\mathrm{S}}}{S_{\mathrm{ASE}}B}\right) = 30.6\,\mathrm{dB}. \tag{S20}$$

We find that the overall transmitter noise level, blue dashed line in Fig. S4, is approximately 5 dB higher compared to pure ASE noise level, green dashed line in Fig. S4.

Based on the in-band noise measurements for the 40 GBd 16QAM single-sideband signals shown in Fig. S4, we estimate the achievable transmitter SNDR for ultra-broadband QAM data signals generated by the OAWG system according to the experimental setup shown in Fig. S5 in Section S4 below. To this end, we make two additional assumptions: First, the SCE is treated as an ideal element in the sense that it merges all tributaries with zero phase error. Second, we assume that the PAPR of the IQ drive signals used to generate the 40 GBd single-sideband signal (Fig. S4) and the IQ drive signals used to generate the 320 GBd QAM waveform (Fig. 5 in the main manuscript) are similar. The $\mathrm{SNDR}_{\mathrm{Tx}}^{(320\,\mathrm{GHz})}$ for a 320 GBd waveform can hence be estimated from the $\mathrm{SNDR}_{\mathrm{Tx}}^{(155.2\,\mathrm{GHz})}$ by accounting for the difference in noise bandwidth.

$$\begin{aligned} \mathrm{SNDR}_{\mathrm{Tx}}^{(320\,\mathrm{GHz})} &\approx \mathrm{SNDR}_{\mathrm{Tx}}^{(155.2\,\mathrm{GHz})} - 10\log_{10}\left(\frac{320\,\mathrm{GHz}}{155.2\,\mathrm{GHz}}\right) \\ &\approx 25.3\,\mathrm{dB} - 3.1\,\mathrm{dB} = 22.2\,\mathrm{dB}. \end{aligned} \tag{S21}$$

Note that the estimated $\mathrm{SNDR}_{\mathrm{Tx}}^{(320\,\mathrm{GHz})}$ may slightly overestimate true transmitter SNDR as we do not record the actual time-domain traces and can therefore not identify all possible signal distortions. Nevertheless, this approach offers the possibility to characterize the signal quality of the OAWG transmitter without including impairments originating from the optical arbitrary waveform measurement (OAWM) receiver as it is, e.g., the case for the transmission experiments presented in Fig. 5 and Fig. 6 in the main manuscript. The estimated transmitter SNDR of 22.2 dB agrees reasonably well with the constellation signal-to-noise ratio (CSNR) of approximately 19 dB measured in our transmission experiments, considering that the CSNR additionally includes noise and distortions from the receiver.

## S3. Constellation signal-to-noise ratio

The constellation signal-to-noise ratio (CSNR) is used in the main manuscript to evaluate the signal quality. The CSNR is defined as the ratio between the average power $P_\mathrm{S}$ of properly normalized ideal symbols with associated complex-valued symbol amplitudes $\underline{S}_{0,n}$ and the average noise-and-distortion power $P_\mathrm{ND}$ contained in the actually received symbols with complex-valued symbol amplitude $\underline{S}_n$,

$$\mathrm{CSNR} = \frac{P_\mathrm{S}}{P_\mathrm{ND}} = \frac{\frac{1}{N_\mathrm{sym}} \sum_{n=1}^{N_\mathrm{sym}} \left|\underline{S}_{0,n}\right|^2}{\frac{1}{N_\mathrm{sym}} \sum_{n=1}^{N_\mathrm{sym}} \left|\underline{S}_n - \underline{S}_{0,n}\right|^2}. \tag{S22}$$

In the above relation, $N_\mathrm{sym}$ is the number of symbols over which the signal and noise-and-distortion powers are averaged. Often, the CSNR is specified in dB, $\mathrm{CSNR_{dB}} = 10\log_{10}(\mathrm{CSNR})$. Note that Eq. S22 requires that the received and transmitted complex-valued symbol amplitudes are normalized and rotated correctly. Further, note that the CSNR is the square of the reciprocal the error vector magnitude (EVM) normalized to the average signal power [5–7]. Both quantities can thus be related by

$$\mathrm{EVM_a} = \sqrt{1/\mathrm{CSNR}}. \tag{S23}$$

## S4. Measurement of 320 GHz signals at different optical signal-to-noise ratios

In Section 2.2.3 of the main manuscript, we presented measurements of 320 GBd quadrature amplitude modulation (QAM) signals at different optical signal-to-noise ratio (OSNR) levels. Figure S5 shows the associated experimental setup along with exemplary optical spectra measured at various points Ⓐ, Ⓑ, Ⓒ, and Ⓔ within the setup. The 320 GBd QAM signals are generated by our four-slice phase-stabilized OAWG system and detected via a two-channel non-sliced OAWM system as described in more detail in Section 2.1 of the main manuscript. We use a dedicated ASE noise source (ASE-C-7-G Desktop ASE Broadband Light Source, Orion Laser Co. Ltd, Shenzhen, China), followed by an EDFA and a variable optical attenuator (VOA) to generate and adjust the ASE power and the associated OSNR of the optical signal that is fed to the OAWM system. Insets Ⓑ and Ⓒ show optical spectra of the generated 320 GBd QAM signal before and after adding different levels of ASE noise, respectively. Inset Ⓓ shows the relation between the attenuation setting of the VOA and the actual OSNR measured at Point Ⓒ using a high-resolution optical spectrum analyzer (Waveanalyzer 1500s, Coherent Corp., Saxonburg, Pennsylvania, USA). Note that the OSNR is measured after a linear polarizer such that only noise that is co-polarized with the signal is considered. To calculate the OSNR from the measured optical PSD $S(f)$ of the data signal, we first calculate the average ASE noise PSD $S_\mathrm{ASE}$ by taking the mean of the noise PSD measured to the left ($S_\mathrm{ASE,l}$) and to the right ($S_\mathrm{ASE,r}$) of the 320 GBd QAM signal spectrum,

$$S_\mathrm{ASE} = \frac{S_\mathrm{ASE,l} + S_\mathrm{ASE,r}}{2}. \tag{S24}$$

Next, the total signal power $P_\mathrm{S}$ is calculated by integrating the PSD $S(f)$ within the signal bandwidth $B_\mathrm{S}$ and by subsequently subtracting the total noise power $P_\mathrm{ASE} = S_\mathrm{ASE} B_\mathrm{S}$ within the signal band,

$$P_\mathrm{S} = \int_{f_\mathrm{cntr} - B_\mathrm{S}/2}^{f_\mathrm{cntr} + B_\mathrm{S}/2} S(f)\mathrm{d}f - S_\mathrm{ASE} B_\mathrm{S}. \tag{S25}$$

For our experiments, the center frequency of the optical signal is $f_\mathrm{cntr} = 192.975\,\mathrm{THz}$, and the total signal bandwidth including a roll-off of $\rho = 1\,\%$ of the RRC pulse-shaping filter amounts to $B_\mathrm{sig} = (1+\rho) \times 320\,\mathrm{GHz} = 323.2\,\mathrm{GHz}$. Finally, the OSNR is calculated by relating the total signal power $P_\mathrm{S}$ to the average noise power within a reference bandwidth $B_\mathrm{ref} = 12.5\,\mathrm{GHz}$ (0.1 nm at a center wavelength of 1550 nm),

$$\mathrm{OSNR_{dB}} = 10\log_{10}\left(\frac{P_\mathrm{S}}{S_\mathrm{ASE} B_\mathrm{ref}}\right). \tag{S26}$$

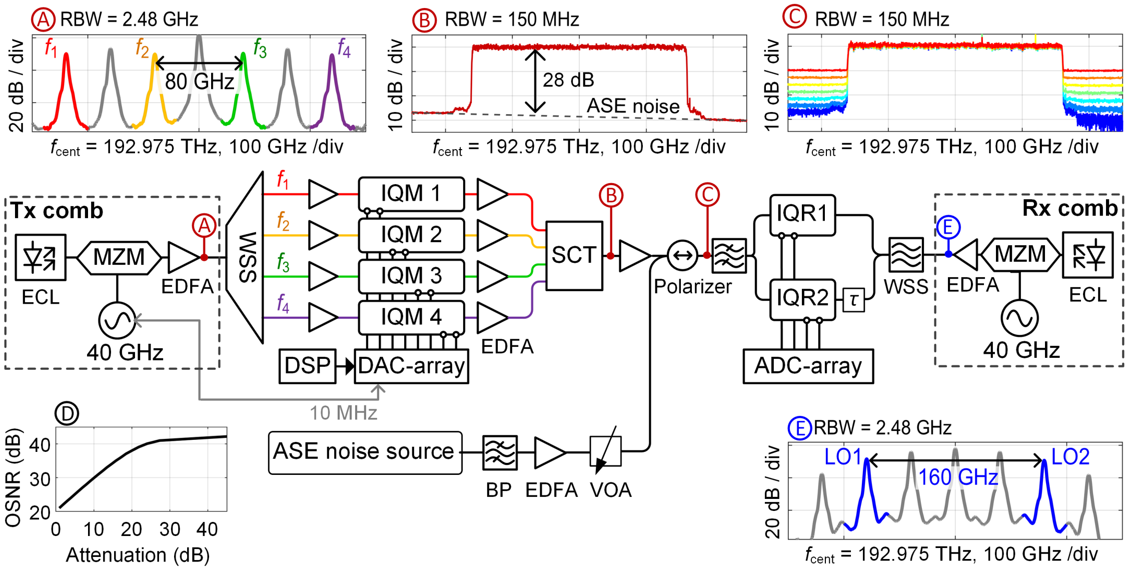


**Fig. S5.** Experimental setup for characterizing the OAWG/OAWM transmission system at different optical signal-to-noise ratio (OSNR) levels along with exemplary measurements taken at points Ⓐ,Ⓑ, Ⓒ, and Ⓔ within the setup. The transmitter comb (Tx comb, Point Ⓐ) is generated by sending a CW tone emitted by an external-cavity laser (ECL) through a Mach-Zehnder modulator (MZM) that is driven by a strong RF signal. The resulting Tx comb is amplified by an erbium-doped fiber amplifier (EDFA), and a wavelength-selective switch (WSS) is used to select individual tones $f_1, f_2, f_3, f_4$ as carriers for IQ modulation. The drive signals for the IQ modulators (IQM1,..., IQM4) are calculated by using offline digital signal processing (DSP) and translated to the analog domain by a DAC array (Keysight M8194A) that is RF-synchronized to the Tx comb generator. A phase-stabilizing signal-combining tree (SCT) combines all tributaries, forming the output waveform $\underline{a}_S(t)$, Point Ⓑ. This waveform is further amplified and loaded with amplified spontaneous emission (ASE) noise, the power of which is controlled by a variable optical attenuator (VOA). The OSNR is measured after a linear polarizer at Point Ⓒ to extract only the co-polarized amplified spontaneous emission (ASE) noise. The resulting noise-loaded waveform is captured by a two-channel non-sliced OAWM receiver. The OAWM system uses two IQ receivers (IQRs) that are fed by the received waveform and by time-delayed copies of a receiver (Rx) comb, comprising two tones (LO1, LO2, Inset Ⓔ). The Rx comb is derived from a second electro-optic (EO) frequency comb that is independent from the transmitter. The photocurrents of the IQRs are digitized by an ADC array (Keysight UXR series oscilloscope). Inset Ⓐ: Optical spectrum of the Tx comb. Note that the displayed spectral width of the individual comb-tones is dictated by the rather large resolution bandwidth (RBW) of the spectrum analyzer that was used for the measurement (RBW = 2.48 GHz). Inset Ⓑ: High-resolution (RBW = 150 MHz) optical spectrum of 320 GBd 16QAM signal. Inset Ⓒ: High-resolution (RBW = 150 MHz) optical spectrum of 320 GBd 16QAM signal after amplification and ASE noise loading. Inset Ⓓ: OSNR levels obtained for various attenuation settings of the VOA. Inset Ⓔ: Optical spectrum of the Rx comb (RBW = 2.48 GHz).

**Funding.** This work was supported by the ERC Consolidator Grant TeraSHAPE (# 773248) and by the associated ERC Proof-of-Concept Grant TeraGear (# 101123567), by the EU H2020 project TeraSlice (# 863322), by the DFG projects PACE (# 403188360) and GOSPEL (# 403187440), by the joint DFG-ANR projects Hybrid-Combs (# 491234846) and Quad- Combs (# 505515860 ), by the DFG Collaborative Research Centers (CRC) WavePhenomena (SFB 1173, # 258734477) and HyPERION (SFB 1527, # 454252029), by the BMBF project Open6GHub (# 16KISK010), by the Horizon Europe EIC transition program with the projects CombTools (# 101136978), MAGNIFY (# 101113302), and HDLN (#101113260), by the Alfried Krupp von Bohlen und Halbach Foundation, by the MaxPlanck School of Photonics (MPSP), and by the Karlsruhe School of Optics & Photonics (KSOP).

## References

1. M. Bachmann, P. A. Besse, and H. Melchior, "General self-imaging properties in N × N multimode interference couplers including phase relations," Appl. optics **33**, 3905–3911 (1994).
2. D. Drayss, D. Fang, C. Füllner, *et al.*, "Non-sliced optical arbitrary waveform measurement (OAWM) using soliton microcombs," Optica **10**, 888–896 (2023).
3. D. Drayss, D. Fang, C. Füllner, *et al.*, "Supplementary document for non-sliced optical arbitrary waveform measurement (OAWM) using soliton microcombs," (2023).
4. D. Drayss, D. Fang, C. Füllner, *et al.*, "Non-sliced optical arbitrary waveform measurement (OAWM) using a silicon photonic receiver chip," J. Light. Technol. **42**, 4733–4750 (2024).
5. S. Forestier, P. Bouysse, R. Quere, *et al.*, "Joint optimization of the power-added efficiency and the error-vector measurement of 20-GHz pHEMT amplifier through a new dynamic bias-control method," IEEE Transactions on Microw. Theory Tech. **52**, 1132–1141 (2004).
6. R. A. Shafik, M. S. Rahman, and A. R. Islam, "On the extended relationships among EVM, BER and SNR as performance metrics," in *International Conference on Electrical and Computer Engineering,* (IEEE, 2006), pp. 408–411.
7. R. Schmogrow, B. Nebendahl, M. Winter, *et al.*, "Error vector magnitude as a performance measure for advanced modulation formats," IEEE Photonics Technol. Lett. **24**, 61–63 (2012).
